# Does attending a structured undergraduate nursing research course affect nursing students’ attitudes toward nursing research? A quasi-experimental study

**DOI:** 10.1371/journal.pone.0351538

**Published:** 2026-07-07

**Authors:** Saleh Al Omar, Islam Ali Oweidat, Khulud Ahmad Rezq, Aziza Salem, Hazem AbdulKareem Alfanash, Khalid Al-Mugheed, Amany Anwar Saeed Alabdullah, Sally Mohammed Farghaly Abdelaliem

**Affiliations:** 1 Faculty of Nursing, Princess Salma Faculty of Nursing, Al al-Bayt University, Al-Mafraq, Jordan; 2 Faculty of Nursing, Zarqa University, Zarqa, Jordan; 3 Community and Psychiatric Health Nursing Department, Faculty of Nursing, University of Tabuk, Tabuk, Saudi Arabia; 4 Faculty of Nursing, University of Tabuk, Tabuk, Saudi Arabia; 5 Faculty of Nursing, Jerash University, Jerash, Jordan; 6 College of Nursing, Riyadh Elm University, Riyadh, Saudi Arabia; 7 Department of Maternity and Pediatric Nursing, College of Nursing, Princess Nourah bint Abdulrahman University, Riyadh, Saudi Arabia; 8 Department of Nursing, Faculty of Allied Health Sciences, Kuwait University, Kuwait, Kuwait; 9 Department of Nursing Administration, Faculty of Nursing, Alexandria University, Alexandria, Egypt; Ajman University, UNITED ARAB EMIRATES

## Abstract

**Aim:**

This study investigated the impact of a structured undergraduate nursing research course on Saudi nursing students’ attitudes toward nursing research.

**Methodology:**

A quasi-experimental pretest-posttest design was used to assess the influence of a 14-week nursing research course at a public Saudi university. Attitudes were measured with a validated questionnaire administered to 62 students before and after the course. A paired t-test was used to evaluate the difference in students’ attitudes toward nursing research.

**Results:**

Students’ attitudes toward nursing research increased significantly after the course (M = 69.58, SD = 9.91) compared to pre-course levels (M = 66.28, SD = 8.43); t(55) = −2.49, p = 0.01). Notable gains occurred in attitudes toward research skills (p = 0.01) and the use of research in clinical practice (p = 0.007). However, there were no statistically significant differences in personal interest in research or in its perceived usefulness. Male students with lower GPAs demonstrated the largest positive attitude shifts.

**Conclusion:**

The research course chiefly enhanced attitudes toward research skills and clinical application, without raising interest or perceived usefulness of nursing research among undergraduate nursing students. Educators should use diverse teaching methods to promote students’ interest in and appreciation of nursing research.

## 1 Introduction

Nursing research occupies a pivotal position in advancing and refining nursing care. It was defined as a systematic and rigorous inquiry utilising diverse methodologies to investigate nursing issues and furnish a scientific foundation for nursing administration, practice, and education [1]. Additionally, nursing research aims to expand the disciplinary knowledge base and enhance health outcomes [2].

Undergraduate nursing students commonly display emotional, cognitive, and behavioural responses toward the nursing profession and research. These are collectively referred to as attitude [3,4]. In nursing, attitude means positive or negative evaluations that may influence future professional conduct and the quality of care. The existing literature often documents favourable attitudes toward research among undergraduate nursing students [5], although some reported negative perceptions of their research abilities [6,7]. A recent study with 603 nursing students showed a moderately positive attitude toward evidence-based research (M = 3.13/5 ± 0.51) [8]. In contrast, some studies reported negative attitudes among nursing students toward research [9–11]. For example, 86% of surveyed nursing students in Vietnam expressed negative research attitudes [12]. In Turkey, 57% of senior nursing students had never read research articles [13]. In Australia, the intention to conduct research lessened among nursing students after attending a research course [14]. In Saudi Arabia, 71% of 186 nursing students found the research course difficult, challenging, and stressful [15]. However, attitudes differ based on instructional context, institutional practices, and cultural factors. Factors that may explain these differences include multicultural awareness [16], cultural competence [17], language proficiency [12], disparities in resources [18–20], research experience, faculty support [19], curriculum design [20], and opportunities for research involvement [21]. These factors could shape nursing students’ attitudes toward research in different regions.

Enhancing nursing students’ interest and participation in research is a critical goal in undergraduate nursing education. Evidence suggests that nursing students gain confidence and improve perceptions when involved in evidence-based projects. Examples include writing group papers, presenting posters [22], writing a thesis [23], joining research courses and summer programs [24], writing research proposals, and critiquing presentations [23,25]. Prior research experience [18] and involvement in data collection [25] can also increase confidence and improve perceptions about research. Although these interventions were effective, limited evidence exists regarding the effects of structured research courses across diverse undergraduate nursing programs.

Nursing faculty play a critical role in cultivating constructive attitudes toward research, thus facilitating research utilisation in clinical practice. Nursing students who receive systematic research training express greater confidence in integrating research evidence into practice [15]. Advancing the nursing profession depends on developing students who value research. Consequently, effective pedagogical strategies for research instruction are vital for improving attitudes. Though research topics are incorporated into undergraduate curricula [23,26] through varied instructional methods [15], more empirical study is needed [27]. While some research-oriented teaching strategies exist, empirical, context-specific guidance for curriculum design is limited. Specifically, the efficacy of structured, in-person, for- credit research courses in Saudi Arabia remains under-investigated, highlighting the need for targeted intervention research that is sensitive to educational and cultural contexts.

It is well-known that nursing education and curricula must use evidence to support learning outcomes. More research is needed to identify the best teaching strategies to improve nursing students’ attitudes toward research [27]. Closing this knowledge gap could guide undergraduate research education. Therefore, this study aimed to examine the impact of a structured undergraduate nursing research course on nursing students’ attitudes at a public university in Saudi Arabia, using a quasi-experimental pretest-posttest design.

## 2 Materials and methods

### 2.1 Study design

This study used a quasi-experimental pretest-posttest design to examine changes in undergraduate nursing students’ attitudes toward nursing research following completion of a structured 14-week undergraduate nursing research course. The structured course included lectures, group discussions, assignments, and case studies that targeted research concepts, skills, and applications. Undergraduate nursing students completed an attitudes questionnaire immediately before and after the course, allowing for within-group comparison of changes over time. Random assignment was not implemented due to logistical restrictions. However, all eligible undergraduate nursing students in the course were included.

### 2.2 Study settings and population

The study was conducted at a nursing faculty within a public university located in northern Saudi Arabia. The faculty began enrolling male and female students seventeen years ago. Currently, 596 students are enrolled across all years. The baccalaureate nursing program encompasses four academic years (eight semesters) followed by a fifth-year internship. All courses are delivered in English.

### 2.3 Study sample

The study sample consisted of all undergraduate nursing students meeting the following inclusion criteria: (a) enrollment in the fourth year, (b) participation in an undergraduate nursing research course, and (c) availability for posttest participation. Exclusion criteria included: (a) enrollment in the first, second, or third year, (b) refusal to participate, and (c) inability to participate in the study. Six students with minimal prior exposure to general research concepts and limited experience with nursing research were included, as their prior experience was informal and lacked structured instruction. Their inclusion reflected the composition of the classroom environment.

G*Power software version 3.1 was used to determine the sample size for the paired t-test [28]. The calculated sample size (n) was 52 participants, based on the following parameters: p = 0.05, power level = 0.8, effect size = 0.4. However, the sample size was augmented by 15% to offset the exclusion of participants who did not meet the inclusion criteria [29]. This helped ensure that the final sample had adequate statistical power despite participant dropout, which is common in pre–post intervention and longitudinal studies. Specifically, the calculated sample size was divided by (1 – anticipated dropout rate). Thus, a total of 62 participants were required for this research. However, six students were excluded from the analysis because they withdrew from the study during the post-test phase.

### 2.4 Intervention

The research in the nursing course was designed to familiarise nursing students with concepts and principles in nursing research, beginning with an introduction to the main stages of the research process. The topics covered were conceptual phase, identifying a research problem, determining of the appropriate research methodology and research instrument, planning for a research study, interpretation and analysis of data, writing conclusions and recommendations, dissemination of research findings, appreciation of the importance of research in the field of nursing, application of evidence-based practice in nursing, and discussion of the various phases of the research process. Both quantitative and qualitative research methods were discussed. The course’s main reference was Essentials of Nursing Research: Appraising Evidence for Nursing Practice [30]. In addition, the students were trained on search strategies to answer questions related to their topic of interest, develop a research proposal, gather pertinent research data, develop a code of behaviours in conducting a systematic study of nursing problems or phenomena, analyse research findings, present their mini-research project, and defend it in class at the end of the semester. It has been reported that participation in scientific research, involvement in data collection, and assistance to researchers can improve the research skills of nursing students [9,11]. Additionally, students submitted an assignment to critique a research study. The educational intervention was a structured, face-to-face, fourteen-week nursing research course in which all students were required to complete learning activities. Student performance was assessed using multiple methods, including written assignments, project-based tasks, and class participation, with research-related tasks. The intervention was deliberately designed to influence attitudes toward nursing research by strengthening research knowledge, improving research literacy, and developing skills for research utilisation. Learning activity, teaching strategies, and assessment methods were aligned to improve students’ research attitudes. A variety of learning activities were used in this course to help students develop their research skills. The students were also given the opportunity to participate in small-scale research activities that allowed them to apply research concepts. A detailed description of the course content is provided in Supplementary File (S1).

To encourage consistency and standardisation of this educational intervention, several procedures were developed. All sections of this course followed a standardised syllabus, course objectives, course materials, and evaluation strategies. Regular meetings for all course instructors were held during this term to standardise course delivery. In addition, all sections used the same assessment techniques, learning activities, and assignments. The standardisation process helped ensure consistency in the educational intervention and internal validity.

### 2.5 Instrument

Students’ attitudes toward nursing research were measured via a self-report validated questionnaire developed by Halabi and Hamdan-Mansour [7]. The scale comprises 22 items and has internal consistency, Cronbach’s alpha of 0.74 [7], 0.86 [14], and 0.84 in the current study, indicating fair to good reliability. It is a four-point Likert scale, where 1 represents “strongly disagree,” and 4 represents “strongly agree.” Each item of this scale has a different dimension in assessing attitudes toward research. The domains of this scale included students’ personal interest in research, the usefulness of research, the use of research in clinical practice, and research abilities. Moreover, the instrument was used in a relevant study [14].

### 2.6 Ethics statement

Ethical approval was obtained from the University of Tabuk research ethics committee (IRB no. UT-236-94-2023). Participants’ anonymity and confidentiality were maintained. They were asked to sign an informed consent form indicating that participation in the study is voluntary. Furthermore, the study was conducted in accordance with the Declaration of Helsinki.

### 2.7 Data collection

The researchers approached the selected setting that approved the data collection. Student lists were screened for eligible participants. After the study was explained and a consent form was signed, a pretest-structured questionnaire was distributed to eligible students prior to the commencement of the research course. This was followed by attending a structured nursing research course. Then, a posttest-structured questionnaire was distributed to the same group of students immediately after completing the 14-week research course and was administered over five days. However, six students withdrew during the posttest; they were excluded from the final analysis, as reported in the results section. The pretest, intervention, and posttest lasted about 4 months, from 30 January 2023–15 May 2023, which corresponds to the length of the semester during which students attended the 14-week course. The study’s total duration lasted 4 months due to limitations in the academic calendar and data-collection processes.

### 2.8 Data analysis

The Statistical Package for Social Sciences (SPSS) version 26 was used for data analysis [31]. Descriptive statistics were used to describe the data. In addition, a paired-samples t-test was employed to analyse students’ attitudes. The level of significance was set at 0.05. The paired-samples t-test provided an internal control for individual differences by using each individual’s baseline attitude as a within-subjects control. Moreover, data were screened for their completeness and coded numerically according to the instrument scoring system. Normality of the attitude scores was assessed using visual inspection of histograms and Q–Q plots, as well as skewness and kurtosis, which indicated acceptable scores for parametric testing. Given the approximate normal distribution, a paired-samples t-test was deemed appropriate.

## 3 Results

### 3.1 Demographic characteristics

The study sample consisted of 62 nursing students. Out of these, six did not complete the post-test and were excluded from the study. Therefore, all inferential statistical analyses were conducted on the final sample of 56. All results, including paired comparisons and correlation analyses, were based on this final sample of 56 students. More than half of the 56 students were females (60.72%) and 21.52 (0.85) years old, with a mean GPA of 4.22 (0.47). Only two students (3.57%) had ever studied an optional research course during their first-year preparatory course. This course was intended for students from all undergraduate programs at the university. It included a brief introduction to the basics of research across disciplines, rather than focusing solely on nursing research. Additionally, eight students (14.29%) were briefly oriented to the research process in previous courses before undertaking the educational intervention, and ten students (17.86%) reported prior use of research concepts. These two groups of students had not previously received information about nursing research. Instead, they were taught general research principles and utilised them in assignments from previous courses (see Table 1).

**Table 1:** Sociodemographic and research-related characteristics among students.

Characteristic	Category	The original sample (N = 62)	The final sample (N = 56)	The original sample (N = 62)	The final sample (N = 56)
		n (%)	n (%)	M (SD)	M (SD)
Age	–	–	–	21.52 (0.86)	21.51 (0.85)
GPA	–	–	–	4.19 (0.47)	4.22 (0.47)
Sex	Male	23 (37.09%)	22 (39.28%)	–	–
	Female	39 (62.91%)	34 (60.72%)	–	–
Have you studied research concepts before?	No	59 (95.16%)	54 (96.43%)	–	–
	Yes	3 (4.84%)	2 (3.57%)	–	–
Where did you study research concepts?	First-year bachelor	2 (66.70%)	2 (100.0%)	–	–
	High school	1 (33.30%)	0 (0.0%)	–	–
Were you introduced to research principles before starting this course?	No	53 (85.48%)	48 (85.71%)	–	–
	Yes	9 (14.52%)	8 (14.29%)	–	–
Where did you learn the research principles before this course?	First-year bachelor	4 (44.44%)	4 (50.00%)	–	–
	High school	4 (44.44%)	3 (37.50%)	–	–
	Workshop	1 (11.12%)	1 (12.50%)	–	–
Have you used research concepts before?	No	50 (80.64%)	46 (82.14%)	–	–
	Yes	12 (19.36%)	10 (17.86%)	–	–
Where did you use research concepts?	Workshop	4 (33.32%)	4 (20.00%)	–	–
	Other courses	8 (66.68%)	6 (60.00%)	–	–

Note, N: Sample Size, M: mean, SD: standard deviation, %: percentage, n: frequency, GPA: Grade Point Average.

### 3.2 Attitudes toward nursing research

The results indicated that students’ overall attitudes score differed significantly after attending the nursing research course, t(55) = −2.49, p = 0.01, 95% CI [−5.97, −0.64]. The attitudes score was significantly higher after attending the research course than before (M = 66.28, SD = 8.43 versus M = 69.58, SD = 9.91) (p = 0.01), with a small-to-moderate standardised effect size (Cohen’s *d* = 0.33). Moreover, the mean total attitude score toward research abilities in the pretest phase was 20.53 (SD = 3.45). However, it increased to 21.96 (SD = 3.72) in the posttest phase, with a statistically significant difference in scores for this domain (p = 0.01). Furthermore, there was a significant improvement in attitudes toward the use of research in clinical practice, with a pretest mean score of 18.39 (SD = 4.26) and a posttest mean score of 20.33 (SD = 4.65) (p = 0.007). However, regarding the domains of research usefulness and personal interest in research, there were no significant statistical differences in scores before and after attending the research course*:* p = 0.89 and p = 0.90, respectively (see Table 2).

**Table 2:** Two-tailed paired samples t-test statistics for the difference in students’ attitudes before and after attending the nursing research course (n = 56).

Subcategory	Pretest M (SD)	Posttest M (SD)	t	p	df	Cohen’s *d*
Overall Scale (22–88)	66.28 (8.43)	69.58 (9.91)	−2.48	0.01	55	0.33
Research Abilities (possible range 7–28)	20.53 (3.45)	21.96 (3.72)	−2.55	0.01	55	0.34
Using Research in Clinical Practice (possible range 7–28)	18.39 (4.26)	20.33 (4.65)	−2.82	0.007	55	0.38
Usefulness of Research (possible range 4–16)	13.91 (1.65)	13.87 (2.06)	.14	0.89	55	0.02
Personal Interest in Research (possible range 4–16)	13.44 (2.12)	13.41 (2.20)	.12	0.90	55	0.02

Note, M: Mean, SD: Standard deviation, t: t-test statistic, p: alpha level of significance, df: Degrees of freedom, Cohen’s *d* = effect size

### 3.3 Differences in attitudes’ scores based on students’ characteristics

**Fig 1:**
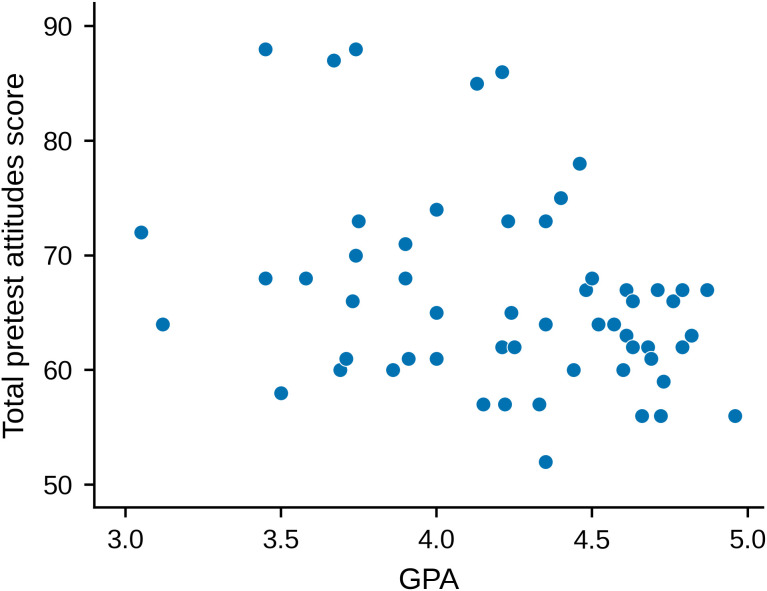
Simple of overall A Grade Point Average.

Sex and the total pretest attitudes score were significantly correlated, *r*(54) = − .31, p = .02. This finding indicates that male students had higher overall attitudes scores toward nursing research than female students did. Moreover, GPA was significantly correlated with the total pretest attitude score (r(54) = − .33, p = .01), indicating that students with higher GPAs had lower attitude scores than those with lower GPAs (see Fig 1).

Nevertheless, no significant correlation was found between GPA and post-test attitudes (r(54) = −0.15, p = 0.10). This finding indicates that the course may have mitigated initial differences in attitudes related to academic achievement and that the 14-week research course helped standardise attitudes across students regardless of their GPAs.

## 4 Discussion

The present study examined the impact of a structured undergraduate nursing research course on undergraduate nursing students’ attitudes toward nursing research. The following section provides a structured discussion of the findings.

### 4.1 Attitudes toward nursing research

The results revealed a significant statistical difference in the mean score of students’ attitudes toward nursing research after they attended the research course (M = 66.28, SD = 8.43 versus M = 69.58, SD = 9.91) (p = 0.01). This finding is comparable to a recent study that showed that, after attending a scientific research education program, the mean attitude score increased from 85.18 ± 9.09 to 90.68 ± 11.16 (p < 0.05) [26]. It was also comparable to findings from a study showing that nursing students who engaged in research activities had more favourable attitudes toward nursing research than those who did not (p < 0.001) [18]. Similarly, the mean attitude score toward research among 56 nursing students increased from 4.49 (0.83) to 5.40 (0.70) after they attended a research course that included instruction on evidence-based practice at a university in Louisiana, United States of America (USA) (p < 0.001) [20]. Specifically, writing critical appraisals and clinical research assignments increased nursing students’ attitudes toward nursing research from 0.59 (SD = 0.08) to 0.85 (SD = 0.14) [20]. However, the prior study lacked a control group and had a small sample size.

The findings contradict the findings of a quasi-experimental study conducted in Australia, which revealed no significant change in nursing students’ attitudes toward research following a research course, and none of the scale domains were significantly different [14]. The mixed findings reported in the literature highlight the importance of pedagogical modality [26]. For example, the Australian study used an online teaching approach [14], which differed from the interactive, face-to-face approach in the present study, suggesting that direct interaction between students and instructors could play an important role in attitude change. In addition, the current findings differ from those of a study conducted in Turkey that reported no statistical difference in students’ attitudes before and after research course completion (U = 3.22; p = 0.33) (n = 86) [32]. These discrepancies could be attributed to variation in the research course, teaching strategies, and educational contexts. Nevertheless, it is important to acknowledge that changing students’ attitudes is expected to occur incrementally over a longer period to manifest in a statistically significant difference.

In the present study, students’ attitude toward nursing research increased by 3.30 points (95% CI: 0.64–5.97), which did not include zero. This indicates that the increase in students’ attitudes toward nursing research was statistically significant, with a magnitude ranging from small to moderate. Accordingly, the effect size was moderate, with Cohen’s d = 0.34, reflecting a small-to-moderate effect and consistent with educational interventions targeting attitudinal outcomes. Improving attitudes is affected by various factors and does not occur quickly. Generally, the course’s delivery and content methods were deliberately designed to improve student engagement, support critical thinking, and strengthen the utilisation of research.

### 4.2 Interpretation of specific sub-scales

Although the improvement in attitude scores is modest, it is statistically significant due to consistent improvements in attitudes towards “research abilities” and “use of research in clinical practice.” Specifically, the score of attitudes toward research abilities increased significantly following completion of the research course (20.53 vs 21.96; p = 0.01), suggesting a positive effect of students’ attitudes toward practical engagement in the research process. In common, hands‑on activities that involve students at each step of the research process might reduce their anxiety about participating in future research and understanding research methodologies [20,22].

A significant increase in students’ attitudes toward using research in clinical practice (18.39 vs 20.33; p = 0.007) illustrates the significance of learning approach contextualisation. In the present study, exposure to interactive learning strategies could enable students to understand the importance of research in real-life situations. The importance of research in bridging the gap between knowledge and practice, while supporting clinical judgement [27], self-efficacy, and cognitive understanding of nursing students [26], is profoundly illustrated by this integration of the research course in the preceding study, which utilised active learning strategies. Mini-projects and article critiques provided students with opportunities to develop research questions, critically appraise previously published studies, and apply the evidence learned in the course to clinical scenarios. Therefore, this type of pedagogical content may explain why students had a significant increase in their attitude toward using research. The increased interaction with research resources and students may be explained in part by structured dialogues and applied learning beyond the classroom, which help students engage in research and positively influence their attitudes toward research in clinical practice.

Students’ scores on the two domains of research usefulness and personal research interest have not statistically improved from before to after attending the research course. This implies that many of the students in this course had their perceptions established before the pre-course intervention and/or were less affected by short-term educational strategies. In other words, the affective part of attitudes could require additional engaging education and training to improve. Future courses may benefit from these results by focusing on research integration rather than solely on cognitive aspects of research. They should also focus on improving research mentorship and facilitating students’ participation in publishing research and presenting findings at conferences, thereby strengthening their personal interest in research and their attitude toward its usefulness.

### 4.3 Differences in attitudes’ scores based on students’ characteristics

A significant correlation was found between GPA and pre-test attitudes toward research (r = −0.33, p = 0.01). However, these findings contradict those of a study that found no significant correlation between students’ GPAs and Saudi nursing students’ attitudes toward research [6]. The negative correlation observed between nursing students who attended nursing research and their GPAs in the present study could be explained by time constraints, greater academic pressure, and the perceived course workload among students with high academic performance [33]. Additionally, the social comparison may influence students’ attitudes [34]. In this study, students with higher GPAs appeared to view the nursing research course as an additional academic burden. They may be more focused on their clinical training and exam preparation than on research. Academic stress may be a contributing factor, but it is possible that academic environments, evaluation methods, and sampling procedures are also influencing this. It is worth noting that this is a relationship with the pre-test attitude scores, not a correlation between GPA and post-test attitude scores. Therefore, future studies are required to provide further insight regarding the implications of this inverse relationship. Specifically, qualitative studies could provide additional insight into the experience underlying this inverse relationship. Nursing academicians are encouraged to adopt strategies to improve the relationships between nursing research, theory, and practice, which could help engage high-performing students in research activities.

Consistent with previous studies, male students scored a higher attitude toward nursing research than female students [12,35]. A study in Nigeria documented that 37% of female nursing students perceived research as stressful and complicated [35]. In contrast, a study conducted in the Philippines found no significant difference in nursing students’ attitudes toward research between males and females (t = −0.007, p = 0.994) [36]. It was also documented that females had less favourable attitudes toward the educational environment than males [37]. In general, cultural and contextual factors vary across countries, which may explain differences in nursing students’ attitudes toward nursing research by students’ characteristics.

### 4.4 Strengths and limitations

The present study is the first to examine the combined effect of various teaching and learning methods in a single course: direct instruction, critiquing research, undertaking mini-research projects, and oral presentations. This approach corresponds to the professional expectations of nursing researchers. Second, the attrition rate was low; only six students did not participate in the posttest. This minimised the risk of attrition bias. Third, the use of a validated research instrument increased the study’s rigour. Despite this, the study has some limitations. Firstly, this study used a convenience sample, which may introduce homogeneity in the sample’s characteristics. There was no control group in this study. This was not possible since all students follow the same course. This limited the ability to make causal inferences and to attribute changes solely to the intervention. Causal interpretations are therefore presented cautiously, emphasising significant changes in attitude as associated with the intervention rather than definitively caused by it. While several measures to enhance internal validity were applied to the evaluation, causality could not be established between the independent and dependent variables due to the lack of a control group. Therefore, the results should be interpreted as associations rather than causation. Third, there was a risk of history and confounding bias. No data were collected on how students were involved in research activities other than the nursing research course in the same semester. These uncontrolled variables could have had a positive or negative effect on attitude levels.

Fourth, testing effects could have occurred in response to the posttest questionnaire, as students might have become aware of content relevant to this study. Fifth, the study was conducted at a single university, limiting the generalisability of the findings. Six, maturation risk, which reflects changes in students over time, such as overall improvement in knowledge and skills, could affect the results. Concurrent coursework and increased academic exposure during the semester. Seventh, while power analysis indicated that the sample size had sufficient power to detect a statistically significant difference between the groups [37], it is recommended that future research be conducted with a larger sample to further enhance the robustness and generalisability of the findings. Finally, the Hawthorne effect and social desirability bias. Students change their behaviour and responses because they may be aware that they are participating in the study, or because they believe such responses are expected or viewed positively. These biases need to be taken into account when interpreting the results. This research was conducted solely to assess students’ attitudes towards nursing research, rather than any other outcomes that may be considered pertinent to this research. Therefore, future research is recommended to include a set of outcome measures to provide a more comprehensive evaluation of the educational intervention.

Although these limitations were unavoidable due to curricular requirements and institutional constraints, measures were taken to reduce them; for example, all course sections followed standardised content, teaching methods, assessments, and delivery. Course instructors convened regularly to ensure consistency across sections and coordinate course delivery. To maintain data integrity and to decrease attrition, data collection was synchronised with course activities.

### 4.5 Implications and recommendations

The findings suggest that, when designing course syllabi, nursing faculty should prioritise interactive research activities to improve students’ engagement. Incorporating facilitated discussions, a mini-research project, and a research critique are recommended. Improving attitudes does not necessarily lead to behavioural change; therefore, future research studies should investigate how positive attitudes toward research could affect actual research involvement and the integration of evidence-based practice. Future studies should investigate a broader range of students’ characteristics, as they could affect students’ attitudes toward research. Additionally, a longitudinal study is recommended to assess the sustainability of changes in attitudes over a longer period. Conducting a multisite study could improve the generalizability of the findings. Moreover, undertaking a mixed-method approach in future studies could provide a deeper understanding of students’ attitudes toward nursing research.

## 5 Conclusions

Attitudes toward nursing research among undergraduate nursing students improved significantly after exposure to a research course, especially in research abilities and the use of research in clinical practice. However, the degree of improvement was moderate. No significant improvement was observed in personal interest in research or in perceived research usefulness. Since the research design was quasi-experimental and lacked a control group, the results should not be considered causal.

The findings could help increase focus on discussions, critiques of scientific articles, and a mini-project to improve nursing students’ attitudes toward research. However, a future randomised controlled trial is recommended to add more rigorous evidence about the impact of other teaching methods on nursing students’ attitudes toward nursing research.

## Supporting information

S1 Table Detailed 14-Week Nursing Research Course Structure (The Educational Intervention). (DOCX)

S2 Data set. (XLSX)
