# Supplementary material for: Does attending a structured undergraduate nursing research course affect nursing students’ attitudes toward nursing research? A quasi-experimental study
Source: PLoS One. 2026 Jul 7;21(7):e0351538. doi: 10.1371/journal.pone.0351538 (PMC13340820; doi:10.1371/journal.pone.0351538)
Supplement: S1 Table — (DOCX) [file pone.0351538.s001.docx]

S1 Table. Detailed 14-Week Nursing Research Course Structure (The Educational Intervention)

| Week | Topic | Learning Objectives | Content/ Activities | Assessment/ Deliverables |
| --- | --- | --- | --- | --- |
| 1 | Introduction to Nursing Research | Students will be able to:  1) Define nursing research.  2) Describe at least three purposes of nursing research.  3) Identify the role of research in evidence‑based practice.  4) Explain the relationship between nursing research and patient outcomes.  5) List major steps in the research process. | Overview of research in nursing, reasons for research, role of research in evidence-based practice | Participation/  Reflection |
| 2 | Ethics in Nursing Research | Students will be able to:  1) State the key ethical principles in research.  2) Identify elements of informed consent.  3) Explain confidentiality and privacy in data collection.  4) Recognise potential ethical dilemmas in research studies.  5) Apply ethical decision-making to case scenarios. | Research ethics, informed consent procedures, confidentiality principles; discussion of ethical dilemmas | Quiz on ethical principles |
| 3 | Identifying a Research Problem | Students will be able to:  1) Distinguish researchable problems from general clinical issues.  2) Construct a clear problem statement.  3) Formulate specific, measurable research questions.  4) Differentiate between research questions and hypotheses.  5) Justify the selection of a nursing research topic. | Steps to identify problems, problem significance, hypotheses, and concept formulation | Draft research question |
| 4 | Literature Review & Database Searching | Students will be able to:  1) Identify appropriate databases for nursing research.  2) Design complex search strategies using Boolean operators.  3) Select relevant keywords and MeSH terms.  4) Evaluate the quality of research articles.  5) Organize literature using reference management software. | Use electronic databases (PubMed, CINAHL, etc.); define keywords; Boolean search strategies | Search log and list of relevant articles |
| 5 | Research Designs & Sampling Strategies | Students will be able to:  1) Compare quantitative and qualitative research designs.  2) Define probability and non-probability sampling methods.  3) Justify the choice of a sampling approach for a given study.  4) List common threats to internal and external validity.  5) Explain how sampling size affects power and attrition. | Types of designs (quantitative, qualitative, mixed); sampling; sample size considerations, including attrition | Diagram of research design choice |
| 6 | Measurement and Instrument Development | Students will be able to:  1) Define reliability and validity in measurement.  2) Differentiate types of reliability.  3) Construct items consistent with theoretical constructs.  4) Assess instrument appropriateness for the study purpose.  5) Apply steps to pilot test an instrument. | Reliability and validity concepts; instrument construction; Likert scaling | Draft instrument items |
| 7 | Data Collection Methods | Students will be able to:  1) Compare structured and unstructured data collection methods.  2) Describe procedures for administering surveys/interviews.  3) Prepare data collection protocols.  4) Identify common biases in data collection.  5) Create a plan to ensure data quality and fidelity. | Structured vs unstructured methods; survey administration; observation | Data collection plan |
| 8 | Quantitative Data Analysis I | Students will be able to:  1) Describe data types and scale levels.  2) Compute measures of central tendency.  3) Interpret measures of variability.  4) Enter and manipulate data in SPSS.  5) Present descriptive results in tables/graphs | Introduction to descriptive statistics; use of SPSS; interpreting output | Descriptive statistics summary |
| 9 | Quantitative Data Analysis II | Students will be able to:  1) State assumptions for parametric tests.  2) Perform basic statistical tests.  3) Interpret significance and confidence intervals.  4) Explain p‑values in context.  5) Distinguish between statistical and clinical significance. | t‑tests, chi‑square, ANOVA concepts; assumptions for parametric testing; hands‑on SPSS | Analysis interpretation report |
| 10 | Qualitative Data Analysis | Students will be able to:  1) Identify procedures for qualitative coding.  2) Develop initial code lists from sample data.  3) Construct thematic categories.  4) Ensure data trustworthiness.  5) Justify methodological choices for analysis. | Coding steps; developing themes; trustworthiness criteria | Codebook draft |
| 11 | Writing Research Findings | Students will be able to:  1) Summarise quantitative results clearly.  2) Present qualitative themes effectively.  3) Select appropriate tables/figures.  4) Maintain APA style in results.  5) Write narrative interpretations aligned with evidence. | Writing results; tables and figures; APA reporting standards | Draft results section |
| 12 | Critical Appraisal Skills | Students will be able to:  1) Identify strengths in research articles.  2) Identify limitations.  3) Evaluate internal/external validity.  4) Apply a selected structured appraisal tool.  5) Submit a written critique (assignment). | Appraisal frameworks; strengths/limitations identification | Critique assignment (submitted this week) |
| 13 | Research Utilisation in Practice | Students will be able to:  1) Define knowledge translation.  2) Identify barriers to research use.  3) Propose strategies to improve utilisation.  5) Reflect on how research improves patient outcomes | Knowledge translation, barriers/facilitators to research use | Written reflection on evidence application |
| 14 | Presentation and Defense | Students will be able to:  1) Defend methodological choices.  2) Summarise key findings.  3) Respond to peer questions.  4) Provide constructive feedback to colleagues. | Oral presentations; questions and answers; peer review; integration of course topics | Final presentation and defence |

Face-to-Face Delivery:
All weeks reflect in-person instruction, discussion, demonstration, and hands-on workshop.

Fig 1: Simple of overall A GPA *


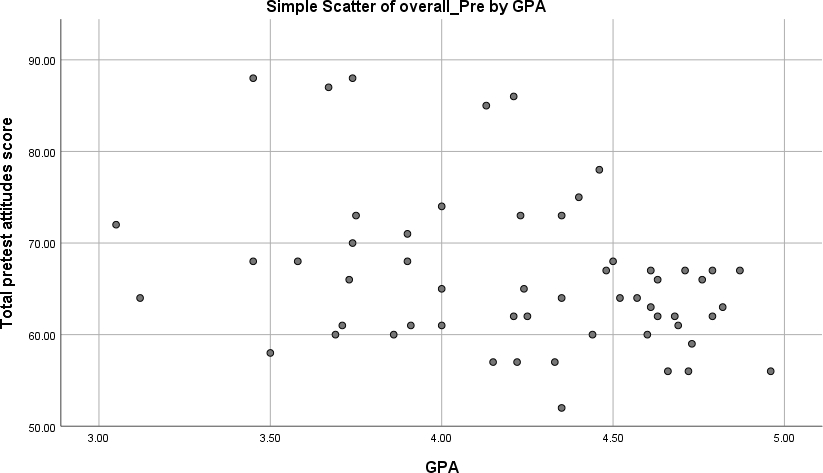


## ^* GPA: Grade Point Average^
