# Supplementary material for: Does attending a structured undergraduate nursing research course affect nursing students’ attitudes toward nursing research? A quasi-experimental study
Source: PLoS One. 2026 Jul 7;21(7):e0351538. doi: 10.1371/journal.pone.0351538 (PMC13340820; doi:10.1371/journal.pone.0351538)
Supplement: Attachment — Submitted filename: PONE-D-25-61156_reviewed on 4th Dec.pdf [file pone.0351538.s003.pdf]

PLOS ONE

Understanding Nursing Students’ Attitudes Toward Research A Quasi-experimental Study

--Manuscript Draft--

**Manuscript Number:**	PONE-D-25-61156
**Article Type:**	Research Article
**Full Title:**	Understanding Nursing Students’ Attitudes Toward Research A Quasi-experimental Study
**Short Title:**	Nursing Students’ Attitudes Toward Research
**Corresponding Author:**	Khalid AL-Mugheed<br>Riyadh Elm University<br>Riyadh, JORDAN
**Keywords:**	Research; attitudes; Course; Nursing
**Abstract:**	Aim<br><br>To assess the influence of a nursing research course on the attitudes of Saudi nursing students toward nursing research.<br><br>Methodology<br><br>A quasi-experimental study was undertaken at a public university in Saudi Arabia. Sixty-two students were enrolled in the current study using a pretest-posttest design. The researchers assessed the effect of enrolment in a nursing research course on students' attitudes toward nursing research. A paired group t-test was used to assess the difference between the mean levels of students’ attitudes toward nursing research.<br><br>Results<br><br>After attending the research course, nursing students’ attitudes increased significantly (M = 69.58, SD = 9.91) compared with their score before the course (M = 66.28, SD = 8.43), t(55) = -2.49, p = 0.01, 95% CI [-5.97, -0.64]. Significant improvement was observed in attitudes towards “research skills” (p = 0.01) and “the use of research in clinical settings” (p = 0.007). Nonetheless, differences in the scores of "personal interest in research" and "usefulness of research" were not statistically significant. Moreover, male students and those with lower grade point averages (GPAs) exhibited more positive attitudes.<br><br>Conclusion<br><br>Attending a nursing research course significantly improved nursing students’ attitudes toward research, especially their perceived research skills and use of research in clinical settings. Nursing education stakeholders are encouraged to facilitate the implementation of various nursing research teaching approaches, as well as supervise the writing of research proposals, research analysis activities, and classroom dialogues.
**Order of Authors:**	Saleh Al Omar
	Islam Ali Oweidat
	Khulud Ahmad Rezq
	Aziza Salem
	Hazem Alfanash
	Khalid AL-Mugheed
	Sally Mohammed Farghaly Abdelaliem
**Additional Information:**	

Powered by Editorial Manager® and ProduXion Manager® from Aries Systems Corporation

Question	Response
**Financial Disclosure**<br><br>Enter a financial disclosure statement that describes the sources of funding for the work included in this submission. Review the submission guidelines for detailed requirements. View published research articles from *PLOS ONE* for specific examples.<br><br>This statement is required for submission and **will appear in the published article** if the submission is accepted. Please make sure it is accurate.<br><br>**Funded studies**<br>Enter a statement with the following details:<br>• Initials of the authors who received each award<br>• Grant numbers awarded to each author<br>• The full name of each funder<br>• URL of each funder website<br>• Did the sponsors or funders play any role in the study design, data collection and analysis, decision to publish, or preparation of the manuscript?<br><br>Did you receive funding for this work?	The author(s) received no specific funding for this work.
**Competing Interests**<br><br>Use the instructions below to enter a competing interest statement for this submission. On behalf of all authors, disclose any competing interests that could be perceived to bias this work—acknowledging all financial support and any other relevant financial or non-financial competing interests.<br><br>This statement is required for submission and **will appear in the published article** if the submission is accepted. Please make sure it is accurate and that any funding sources listed in your Funding Information later in the submission form are also	The authors have declared that no competing interests exist.

declared in your Financial Disclosure statement.<br><br>View published research articles from *PLOS ONE* for specific examples.<br><br>**NO authors have competing interests**<br><br>Enter: *The authors have declared that no competing interests exist.*<br><br>**Authors with competing interests**<br><br>Enter competing interest details beginning with this statement:<br><br>*I have read the journal's policy and the authors of this manuscript have the following competing interests: [insert competing interests here]*<br><br>* typeset	
**Ethics Statement**<br><br>Enter an ethics statement for this submission. This statement is required if the study involved:<br><br>• Human participants<br>• Human specimens or tissue<br>• Vertebrate animals or cephalopods<br>• Vertebrate embryos or tissues<br>• Field research<br><br>Write "N/A" if the submission does not require an ethics statement.<br><br>General guidance is provided below. Consult the submission guidelines for detailed instructions. **Make sure that all information entered here is included in the Methods section of the manuscript.**	. The study was approved by the Institutional Review Board at the University of Tabuk (UT-236-94-2023). They were asked to sign written informed consent, indicating that participation in the study is voluntary.

Powered by Editorial Manager® and ProduXion Manager® from Aries Systems Corporation

**Format for specific study types**<br><br>**Human Subject Research (involving human participants and/or tissue)**<br>• Give the name of the institutional review board or ethics committee that approved the study<br>• Include the approval number and/or a statement indicating approval of this research<br>• Indicate the form of consent obtained (written/oral) or the reason that consent was not obtained (e.g. the data were analyzed anonymously)<br><br>**Animal Research (involving vertebrate animals, embryos or tissues)**<br>• Provide the name of the Institutional Animal Care and Use Committee (IACUC) or other relevant ethics board that reviewed the study protocol, and indicate whether they approved this research or granted a formal waiver of ethical approval<br>• Include an approval number if one was obtained<br>• If the study involved *non-human primates*, add *additional details* about animal welfare and steps taken to ameliorate suffering<br>• If anesthesia, euthanasia, or any kind of animal sacrifice is part of the study, include briefly which substances and/or methods were applied<br><br>**Field Research**<br><br>Include the following details if this study involves the collection of plant, animal, or other materials from a natural setting:<br>• Field permit number<br>• Name of the institution or relevant body that granted permission	
**Data Availability**<br><br>Authors are required to make all data underlying the findings described fully available, without restriction, and from the time of publication. PLOS allows rare exceptions to address legal and ethical concerns. See the PLOS Data Policy and FAQ for detailed information.	Yes - all data are fully available without restriction

A Data Availability Statement describing where the data can be found is required at submission. Your answers to this question constitute the Data Availability Statement and **will be published in the article**, if accepted.<br><br>**Important:** Stating 'data available on request from the author' is not sufficient. If your data are only available upon request, select 'No' for the first question and explain your exceptional situation in the text box.<br><br>Do the authors confirm that all data underlying the findings described in their manuscript are fully available without restriction?	
**Describe where the data may be found in full sentences. If you are copying our sample text, replace any instances of XXX with the appropriate details.**<br><br>• If the data are **held or will be held in a public repository**, include URLs, accession numbers or DOIs. If this information will only be available after acceptance, indicate this by ticking the box below. For example: *All XXX files are available from the XXX database (accession number(s) XXX, XXX.*).<br>• If the data are all contained **within the manuscript and/or Supporting Information files**, enter the following: *All relevant data are within the manuscript and its Supporting Information files.*<br>• If neither of these applies but you are able to provide **details of access elsewhere**, with or without limitations, please do so. For example:<br><br>*Data cannot be shared publicly because of [XXX]. Data are available from the XXX Institutional Data Access / Ethics Committee (contact via XXX) for researchers who meet the criteria for access to confidential data.*<br><br>*The data underlying the results presented in the study are available from (include the name of the third party*	The data that support the findings of this study are available on request from the corresponding author.

Powered by Editorial Manager® and ProduXion Manager® from Aries Systems Corporation

and contact information or URL).<br>• This text is appropriate if the data are owned by a third party and authors do not have permission to share the data.<br><br>* typeset	
Additional data availability information:	

Powered by Editorial Manager® and ProduXion Manager® from Aries Systems Corporation

Manuscript
Click here to access/download;Manuscript;Manuscript Research Course Quasi with author details.docx

Understanding Nursing Students' Attitudes Toward Research A Quasi-experimental Study

Saleh Al Omar [1], Islam Ali Oweidat [2], Khulud Ahmad Rezq [3], Aziza Salem [4], Hazem Alfanash [5] Khalid Al-Mugheed [6], Sally Mohammed Farghaly Abdelaliem [7]

[1] Saleh Al Omar, RN, Ph.D. Assistant Professor, Faculty of Nursing, Princess Salma Faculty of Nursing, Al al-Bayt University, P.O. BOX 130040, Al-Mafraq 25113, Jordan ORCID ID: https://orcid.org/0000-0002-4495-1030 Mobile No: +962-776114184 Tel: +962-2-6297000 Fax: +962-2-6297053 E-mail: salomar@aabu.edu.jo.

[2] (Assistant Professor, Faculty of Nursing, Zarqa University, Zarqa, Jordan)
P.O Box number: 13110 e-mail address: ioweidat@zu.edu.jo
ORCID ID: https://orcid.org/0000-0003-2344-4299

[3] (Assistant Professor, Faculty of Nursing, University of Tabuk, Tabuk, Saudi Arabia
P.O Box number: 71491 e-mail address: krizk@ut.edu.sa

[4] Assistant Professor, Faculty of Nursing, University of Tabuk, Tabuk, Saudi Arabia
P.O Box number: 71491 e-mail address: asalem@ut.edu.sa
ORCID ID: https://orcid.org/0000-0003-2955-0172

[5] (Assistant Professor, Faculty of Nursing, University of Tabuk, Tabuk, Saudi Arabia
P.O Box number: 71491 e-mail address: halfanash@ut.edu.sa
ORCID ID: https://orcid.org/0000-0003-2168-2525

6 Assistant Professor of nursing /College of Nursing, Riyadh Elm University, Riyadh, Saudi Arabia. (khalidanwer.almugeed@neu.edu.tr).

7- Associate professor, Department of Nursing Management and Education, College of Nursing, Princess Nourah bint Abdulrahman University, P.O. Box 84428, Riyadh 11671, Saudi Arabia Riyadh. (smfarghaly@pnu.edu.sa)

Corresponding author:
Saleh Al Omar
Assistant Professor, Faculty of Nursing, Princess Salma Faculty of Nursing, Al al-Bayt University, P.O. BOX 130040, Al-Mafraq 25113, Jordan ORCID ID: https://orcid.org/0000-0002-4495-1030 Mobile No: +962-776114184 Tel: +962-2-6297000 Fax: +962-2-6297053 E-mail: salomar@aabu.edu.jo

Understanding Nursing Students' Attitudes Toward Research
A Quasi-experimental Study

Abstract

Aim: To assess the influence of a nursing research course on the attitudes of Saudi nursing students toward nursing research.

Methodology: A quasi-experimental study was undertaken at a public university in Saudi Arabia. Sixty-two students were enrolled in the current study using a pretest-posttest design. The researchers assessed the effect of enrolment in a nursing research course on students' attitudes toward nursing research. A paired group t-test was used to assess the difference between the mean levels of students' attitudes toward nursing research.

Results: After attending the research course, nursing students' attitudes increased significantly ($M = 69.58$, $SD = 9.91$) compared with their score before the course ($M = 66.28$, $SD = 8.43$), $t(55) = -2.49$, $p = 0.01$, 95% CI [-5.97, -0.64]. Significant improvement was observed in attitudes towards "research skills" ($p = 0.01$) and "the use of research in clinical settings" ($p = 0.007$). Nonetheless, differences in the scores of "personal interest in research" and "usefulness of research" were not statistically significant. Moreover, male students and those with lower grade point averages (GPAs) exhibited more positive attitudes.

Conclusion: Attending a nursing research course significantly improved nursing students' attitudes toward research, especially their perceived research skills and use of research in clinical settings. Nursing education stakeholders are encouraged to facilitate the implementation of various nursing research teaching approaches, as well as supervise the writing of research proposals, research analysis activities, and classroom dialogues.

Keywords: Research; Attitudes; Course; Nursing

1. Introduction

Nursing research plays an essential role in advancing nursing care. It aims to develop the nursing body of knowledge (1). It was documented that undergraduate nursing students have favourable attitudes toward research (2). However, they had negative attitudes toward their research abilities (3, 4). Other studies reported negative attitudes towards research by nursing students (5-7). In a sample of 186 nursing students in the Kingdom of Saudi Arabia (KSA), 71% viewed research as a difficult, challenging, and stressful subject (8). Similarly, 85.7% of nursing students in Vietnam had negative attitudes toward research difficulty (9). Furthermore, 57% of senior nursing students had never read research articles, as reported in a study conducted in Turkey (10). The intention of nursing students to conduct research decreased after they attended a research course in Australia (11). This difference in the level of attitudes towards nursing research in different countries could vary due to variations in teaching contexts and cultural factors. Some factors that could explain this difference are multicultural awareness (12), cultural competence (13), and language proficiency (9).

Increasing students' interest and participation in research is one of the most important aspects of undergraduate nursing education. Nursing students showed improved confidence and perceptions toward research when an evidence-based project that included writing group papers, poster presentations (14), writing a thesis (15), enrolling in research courses and summer programs (16), writing research proposals (15, 17), having prior research experience (18), critique presentations, and participating in data collection was used (17).

Nursing faculty play an important role in enhancing nursing students' attitudes toward research. Students who were well trained in research were more confident in the use of research-based evidence in nursing practice (8). The development of the nursing profession requires students who are aware of and value research. Therefore, choosing an appropriate method of teaching research is vital for improving attitudes toward nursing research.

Although undergraduate nursing programs usually have nursing research courses in their curricula (19), different methods and approaches are used to teach these courses (8). Different research teaching strategies have been reported in the literature and studied in isolation; however, more evidence is still needed (1). Improving students' attitudes toward research is crucial, as it could improve the utilisation of research findings.

Nursing education and curricula must be evidence-based to improve learning outcomes. Additional research studies are required to determine the appropriate learning and teaching strategies to positively influence nursing students' attitudes toward research (1). Therefore, it is crucial to find solutions that improve the attitudes of nursing students toward research. This may help close this gap in research literature and meaningfully redirect the path of undergraduate research education. Therefore, this study aimed to assess the effect of a nursing research course on Saudi nursing students' attitude toward nursing research at a public university in the KSA.

2. Methods:

2.1 Study Design

This study employed a quasi-experimental pretest–posttest approach to assess differences in nursing students' attitudes towards research following enrolment in a

structured 14-week nursing research course. This design was previously used to investigate relevant research problems (14, 15, 20). It can be used to evaluate the outcomes and effectiveness of educational or clinical interventions at two stages: before and after the intervention, using the same group of participants, where random assignment is not possible (21).

2.2 Study Settings and Population

The study was conducted at a faculty of nursing at a public university in northern Saudi Arabia. Sixteen years ago, this faculty has enrolled students of both sexes and has 596 students across different years of study. The baccalaureate nursing program is four academic years, divided into eight semesters, followed by a fifth year for an internship period. Moreover, the language of teaching is English.

2.3 Samples and sampling technique

The study sample included all undergraduate nursing students who agreed to take part in the study and fulfilled the eligibility criteria, including being in the fourth year of study, being enrolled in a nursing research course, and being able to participate in the study until the posttest stage. The exclusion criteria were as follows: being in the first year, second year, or third year, refusing to participate in the study, and being unable to participate.

G*Power software version 3.1 was used to determine the sample size for the t-test (22). P and power levels were set at 0.05 and 0.80, respectively. For the paired t-test with an effect size (ES) of 0.4, the calculated sample size (n) was 52 participants. However, the sample size was augmented by 20% to offset the exclusion of participants who do not meet the inclusion criteria. Therefore, the required total sample size was (N=62).

2.4 Intervention

The research in the nursing course was designed to familiarise nursing students with concepts and principles in nursing research, beginning with an introduction to the main stages of the research process. The topics covered were conceptual phase, identifying a research problem, determining of the appropriate research methodology and research instrument, planning for a research study, interpretation and analysis of data, writing conclusions and recommendations, dissemination of research findings, appreciation of the importance of research in the field of nursing, application of evidence-based practice in nursing, and discussion of the various phases of the research process. Both quantitative and qualitative research methods were discussed. In addition, the students were trained on search strategies to answer questions related to their topic of interest, develop a research proposal, gather pertinent research data, develop a code of behaviours in conducting a systematic study of nursing problems or phenomena, analyse research findings, present research output and defend it in class at the end of the semester. It has been reported that participation in scientific research, being involved in data collection, and helping researchers improve the research skills of nursing students (5, 7). Additionally, students submitted an assignment to critique a research study. The course's main reference was Essentials of Nursing Research: Appraising Evidence for Nursing Practice (21). The course was a direct (face-to-face) course that lasted 14 weeks. The lecturers met weekly to ensure the consistency of the teaching methods. The course syllabus, teaching methods, assessment methods, evaluation methods, and assignments were unified across all the course sections. The purpose of these meetings was to synchronise teaching strategies, review course advancement, and avoid

discrepancies between different course sections. This unified strategy enabled a uniform presentation of the intervention, thereby improving the internal validity of the study.

2.5 Instrument

Students' attitudes toward nursing research were measured via a self-report questionnaire developed by Halabi and Hamdan-Mansour (4). The scale is composed of 22 items and has a Cronbach's alpha value of 0.74, indicating fair to good reliability (4), 0.86 (11), and 0.84 in the current study. It is a four-point Likert, where one represents" strongly disagree" and four represents "strongly agree". Each item of this scale has a different dimension in assessing attitudes toward research. The domain of this scale included the students' personal interest in research, the usefulness of research, the use of research in clinical practice, and research abilities. The instrument was used in a relevant study (11).

2.6 Ethics Statement

The ethical approval was obtained by research ethical committee form university of Tabuk with IRP no: (UT- 236-94-2023). Participants gave written informed consent to participate in the study before taking part.

2.7 Data collection

The researchers approached the selected setting that approved the data collection. Lists of students seeking eligible participants were screened. After the study was explained and a consent form was signed, a pretest questionnaire was distributed to the eligible students. This was followed by attending a "nursing research course." Then, a posttest questionnaire was distributed to the same group of students. The process lasted five months, between 30 January 2023 and 15 May 2023.

2.8 Data analysis

The Statistical Package for the Social Sciences (SPSS) version 23 was used to analyse data (23). Descriptive statistics were used to describe the sample characteristics. Moreover, a paired samples t-test was employed to assess the difference between the means of students' attitudes, with a significance threshold set at $\alpha = 0.05$.

3. Results

3.1 Demographic characteristics

The study enrolled 62 nursing students. However, six withdrew from the study. Thirty-nine students were females (62.91%), and their mean age was 21.52 (0.86) years, with a mean Grade Point Average (GPA) of 4.19 (0.47) for the whole sample. Data from 56 fourth-year nursing students were analysed because six students did not complete the posttest assessment. More than half of the 56 students were females (60.7%) and 21.52 (0.85) years old, with a mean GPA of 4.22 (0.47). Only two students (3.57%) had ever studied an optional research course during their first-year preparatory course. This course was intended for students from all undergraduate programs at the university. It included a brief introduction to the basics of research in different disciplines rather than focusing on nursing research. Additionally, eight students (14.29%) were oriented briefly to the research process in previous courses before undertaking the educational intervention, and ten students (17.86%) reported previous utilisation of research concepts. These two groups of students had not previously received information about nursing research. Instead, they were taught about the principles of research in general and utilised these principles in assignments from previous courses (see Table 1).

3.2 Attitudes toward Nursing Research

The results indicated that the overall mean score of students' attitudes differed significantly after they attended the nursing research course, t(55) = -2.49, p = 0.01, 95% CI [-5.97, -0.64]. The attitudes score was significantly higher after attending the research course than before (M=66.28, SD=8.43 versus M=69.58, SD=9.91) (p = 0.01). Specifically, the difference was significant for the following two domains of the scale only: research abilities (p = .01) and using research in clinical practice (p = .007). Nevertheless, the difference was nonsignificant for the domains of personal interest in research and usefulness of research.

The mean total attitudes score toward research abilities in the pretest phase was 20.53 (SD=3.45). However, it increased to 21.96 (3.72) in the posttest phase, with a significant statistical difference in scores of this domain (p=0.01). Moreover, there was a significant improvement in attitudes toward using research in clinical practice, with a pretest mean score of 18.39 (4.26) and a posttest mean score of 20.33 (4.65) (p=0.007). However, regarding the domains of the usefulness of research and personal interest in research, there were no significant statistical differences between scores before and after attending the research course (p=.89 and p=.90, respectively) (see Table 2).

.

3.3 Differences in Attitudes Scores Based on Students Characteristics

Sex and the total pretest attitudes score were significantly correlated (r=-0.31, p=0.02). This indicate that male students had higher overall attitudes scores toward nursing research than female students did. Moreover, GPA was also significantly correlated with the total pretest attitudes score (r=-0.33, p= 0.01). Meaning that students who achieved higher GPAs had lower attitude scores than students with lower GPAs.

4. Discussion

The results of the present study revealed a significant statistical difference in the mean score of students' attitudes toward nursing research after they attended the research course (M=66.28, SD=8.43 versus M=69.58, SD=9.91) (p=0.01). This finding is comparable to the findings of a recent study that showed that after attending a scientific research education program, the mean attitudes score increased from 85.18 ± 9.09 to 90.68 ± 11.16 (p <0.05) (24). This finding was also comparable to the findings of a study conducted by Ünver, Semerci (18), which showed that nursing students who engaged in research activities had more favourable attitudes toward nursing research than students who did not (p<0.001). Similarly, the mean attitudes score for research among nursing students increased from 4.49 (0.83) to 5.40 (0.70) (p<0.001) after they attended a research course that included teaching about evidence-based practice at a university in Louisiana in the United States of America (USA) (n=56) (20). Specifically, writing critical appraisals and clinical research assignments increased nursing

students' attitudes toward nursing research from 0.59 (SD=0.08) to 0.85 (SD=0.14) (20). However, the prior study lacked a control group and had a small sample size.

The findings contradict the findings of a quasi-experimental study conducted in Australia, which revealed no significant change in nursing students' attitudes toward research following a research course, and none of the scale domains were significantly different (11). Nevertheless, the method of research course instruction was online, which differed from the method of course instruction in the present study. This suggests a potential benefit of using interactive and in-class instruction to improve students' attitudes via a face-to-face approach that could increase students' engagement. Additionally, the findings contradict those of a study carried out in Turkey and documented that the difference in the attitudes score was not significant between students before and after attending a research course (U=3.22; p=0.33) (n=86) (25). These contradictory findings could be explained by the differences in the educational contexts and teaching strategies used in each research course.

Although the difference in attitude scores of the present study is mild, it is still statistically significant given the consistent increase in the attitudes scores in the following domains: research abilities (p=0.01) and using research in clinical practice (p=0.007). The effect size (Cohen's d = 0.34) is in the small to moderate range, indicating a significant impact of the intervention. A change in attitudes is expected to occur gradually and requires additional time to show a significant difference. Specifically, the scores of the 'research abilities' domain increased significantly after attending the research course (20.53 vs. 21.96; p =0.01), which signifies the effect of practical engagement. Engaging students in every step of the research process and using hands-on activities in these courses might reduce their anxiety as they think about research methodologies and future research involvement (14, 20). In addition, improving the mean score

of students' attitudes toward using research in clinical practice (18.39 vs. 20.33; p=0.007) signifies the importance of adapting learning to the available context. In the present study, students were exposed to interactive learning and research projects, which could enable them to notice the value of research in real-life situations. This highlights the significance of utlising research to support the link between practice and knowledge, which could further improve clinical judgment (1).

In terms of practical level, the improvement in the attitudes level, especially in the domains: using research in clinical practice and research abilities, indicated that attending the nursing research course improved students' confidence in their ability to utilise research in real-world scenarios, which is congruent with the goals of evidence-based nursing education. It is well known that developing students' skills could support and improve patient care. The research course in the present study involved different strategies, such as mini-projects, critiquing articles, in-class teaching, and discussions. It has been reported that bridging theoretical content with real-world practice could reinforce cognitive understanding and confidence in personal abilities among nursing students (24).

The scores of the domains: usefulness of research and personal research interest did not differ significantly after the students attended the research course. This finding shows that such an affective part of attitudes could require additional engaging education and training to improve. Future courses may benefit from these results, focusing on research integration rather than the cognitive aspects or research only. They should also focus on improving mentorship and facilitating students' participation in publishing research and presenting findings at conferences.

A significant correlation was found between GPA and attitudes toward research (r = -0.33, p = 0.01). However, these findings contradict the findings of a study that revealed no

significant correlation between students' GPA and Saudi nursing students' attitudes toward research (3). The negative correlation between nursing students who attended nursing research and their GPA in the present study could be explained by time constraints, greater academic pressure, and course workload perceived among students with high academic performance (26). Furthermore, the pattern of social comparison can affect students' attitudes (27). In the present study, students with higher GPAs might have perceived the nursing research course as an extra burden. They might prefer to focus more on performance on exams and clinical training than on research. Academicians are encouraged to consider future strategies focusing on the relationships among research, theory, and practice, which could help engage students with high GPA in research activities. Nevertheless, future qualitative studies could help explore the underlying experience that could lead to this inverse relationship.

Male students reported a higher attitude toward nursing research than female students. This was in line with the previous studies (9, 28). Researchers in Nigeria noted that 37% of female nursing students perceived research as complicated and stressful (28). However, these findings contradict those of a study conducted in the Philippines, which revealed that sex has no significant effect on the attitudes of nursing students toward research ($t=-.007$, $p=0.994$) (29). It was documented that females had less favourable attitudes toward the educational environment than males (Chandana & Hettiarachchi, 2013). In general, some cultural and contextual factors could differ from one country to another, which could explain the difference in nursing students' attitudes toward nursing research based on their different characteristics.

Strengths and Limitations

The present study is the first to study the combined effect of various teaching and learning methods in one course: direct instruction, critiquing research, undertaking mini-research

projects, and oral presentation. This approach mirrors the professional expectations of nursing researchers. Second, the attrition rate was low; only six students did not participate in the posttest. This minimised the risk of attrition bias. Third, the use of a validated research instrument increased the study rigour. Nevertheless, some limitations of the study include: First, the sample was convenient, which could lead to homogeneity in terms of the characteristics of the participants and limited representation of students studying at other universities. Second, the study design lacked a control group; this approach was not feasible because all the students had the same program curriculum. This limited the ability to make causal inferences. Third, there was a risk of history and confounding bias. No data were collected concerning students' participation in research activities other than the nursing research course during the same semester. These unmeasured variables could affect the attitudes level positively or negatively. Fourth, the study was conducted at one university only, limiting the generalizability of the findings. Fifth, maturation risk, which reflects changes in students over time, such as overall improvement in knowledge and skills, could affect the results. Sixth, the Hawthorne effect and social desirability bias. Students change their behaviour and responses as they are possibly aware of participating in the study or believe such responses are expected or viewed positively. Some students might express positive attitudes toward research because of these risks. These biases should be considered when interpreting the results, as they could limit the generalizability of the findings. Finally, testing effects could have affected how students responded to the posttest questionnaire, as they might have become more aware of the content relevant to this research study.

Implications and recommendations

The study findings imply that nursing faculty should focus on methods that increase students' engagement in research through research activities while designing nursing curricula and syllabi. On the basis of the study findings, different methods of teaching research courses, such as oral discussions, undertaking a mini-research project, critiquing a research article, and instructing students about different aspects of nursing research, are recommended to improve students' attitudes towards research.

It is essential to consider the practical implications of statistically significant improvements in nursing students' attitudes toward research. Such improvement can be useful for improving students' engagement in research initiatives, their involvement in clinical research activities, the application of evidence-based practices, and the improvement of patient outcomes. However, improving attitudes does not necessarily lead to behavioral change; therefore, future research studies should investigate how positive attitudes toward research could affect research involvement and the integration of evidence-based practice.

A future randomised controlled trial is recommended to add more rigorous evidence about the impact of other teaching methods on nursing students' attitudes toward nursing research. Future studies should investigate a broader range of students' characteristics, as they could affect students' attitudes toward research. Additionally, a longitudinal study is recommended to assess the sustainability of changing attitudes over longer time. Conducting a multisite study could improve the generalizability of the findings. Moreover, undertaking a mixed-methods approach in future studies could provide a deeper understanding of students' attitudes toward nursing research.

5. Conclusions

Nursing students showed a significant improvement in their attitudes towards nursing research after attending a research course, particularly in the domains of research skills and applying research in clinical settings. The fining could aid in increasing the focus on using discussions, critiques of scientific articles, and undertaking a mini-project to improve nursing students' attitudes toward research. However, conducting additional studies using different teaching methods for nursing research courses and different methodologies is recommended.

Declarations

Ethics approval and consent to participate

The study was approved by the Institutional Review Board at the University of Tabuk (UT-236-94-2023). Participants' anonymity and confidentiality were maintained. They were asked to sign informed consent, indicating that participation in the study is voluntary. The study was conducted according to the guidelines of the Declaration of Helsinki and approved by the Institutional Review at all sites.

Consent for publication: Not Applicable

Data Availability Statement: The data that support the findings of this study are available on request from the corresponding author.

Funding: The research study received funding from Princess Nourah bint Abdulrahman University Researchers Supporting Project number (PNURSP2025R444), Princess Nourah bint Abdulrahman University, Riyadh, Saudi Arabia.

Author contribution: SAO, IAO, and KAR performed the literature search, collected and interpreted the data. AS, HA, KAM, and SMFA drafted the work and contributed to the writing of this manuscript. SAO, IAO, KAR, and SMFA edited and drafted the final version of this manuscript. SAO, IAO, KAR, and HA reviewed the final version to be published.

Acknowledgment: The researchers gratefully acknowledge Princess Nourah bint Abdulrahman University Researchers Supporting Project number (PNURSP2025R444), Princess Nourah bint Abdulrahman University, Riyadh, Saudi Arabia.

References

1. Ross JG, Burrell SA. Nursing students' attitudes toward research: An integrative review. *Nurse Educ Today*. 2019;82:79–87. https://doi.org/10.1016/j.nedt.2019.05.022
2. Wanling W, Shuying Z. Chinese nurses' and nursing students' attitudes towards nursing research and its associated factors: A literature review study. 2024.
3. Halabi JO. Attitudes of Saudi nursing students toward nursing research. *Saudi J Health Sci*. 2016;5(3):118. https://doi.org/10.4103/2278-0521.192628
4. Halabi JO, Hamdan-Mansour A. Attitudes of Jordanian nursing students towards nursing research. *J Res Nurs*. 2012;17(4):363–73. https://doi.org/10.1177/1744987110379547
5. Silvia GN, Contreras-Higuera W, Canet-Vélez O, Torralbas-Ortega J, Mercé TA, Judith RO. The effect of writing a bachelor thesis on attitude towards nursing research and development: A cross-sectional comparative study between students and professionals. *Nurse Educ Toda*. 2022 Nov 1;118:105532.
6. Moya-Salazar J, Ccorahua M, Goicochea-Palomino EA, Moya-Espinoza JG, Contreras-Pulache H. Favorable attitude toward research in nursing students during internship: A cross-sectional study in Peru. *SAGE Open Nurs*. 2023;9:23779608231206776. https://doi.org/10.1177/23779608231206776
7. Marendić M, Bokan I, Matana A, Orlandini R, Širić A, Puljak L, et al. Attitudes and factors that affect Croatian nursing students' choice of thesis type: A cross-sectional study. *Nurse Educ Today*. 2023;121:105664. https://doi.org/10.1016/j.nedt.2023.105664
8. Al Furaikh S, Al Omairi B, Ganapathy T. A cross-sectional survey on nursing students' attitude towards research. *J Health Spec*. 2017;5(4):185. https://doi.org/10.4103/jhs.JHS_21_17
9. Van Nguyen T, Nguyen HXT, Nguyen HT, Dao LYT, Nguyen NTT, Pham KBT, et al. Factors associated with attitude toward scientific research: Vietnam nursing students—a cross-sectional study. *Open Access Maced J Med Sci*. 2023;11(G):63–8. https://doi.org/10.3889/oamjms.2023.11725
10. Karaahmetoğlu GU, Kütahyalioğlu NS. Registered nurses' and nursing students' attitudes towards scientific research: A cross-sectional study. *J Nursol*. 2024;27(1):79–87. https://doi.org/10.14744/jon.2023.34761
11. Ramsay A, Wicking K, Yates K. In what ways does online teaching create a positive attitude towards research in nursing students studying a first year evidence-based practice undergraduate subject online? *Nurse Educ Pract*. 2020;44:102744. https://doi.org/10.1016/j.nepr.2020.102744
12. Kardas U, Sahin SY. Investigation of the relationship between cultural sensitivity and effectiveness levels among nursing students. *Nurse Educ Pract*. 2023;72:103773. https://doi.org/10.1016/j.nepr.2023.103773
13. Farooqui S, Jabeen H, Salman M, Kaukab SR. Cultural competence among nursing students in Pakistan: A literature review. *Soc Sci Rev Arch*. 2024;2(2):1573–82.
14. Keib CN, Cailor SM, Kiersma ME, Chen AM. Changes in nursing students' perceptions of research and evidence-based practice after completing a research course. *Nurse Educ Today*. 2017;54:37–43. https://doi.org/10.1016/j.nedt.2017.04.004

15. Toraman A, Hamaratçılar G, Tülü B, Erkin Ö. Nursing students' attitudes toward research and development within nursing: Does writing a bachelor thesis make a difference? *Int J Nurs Pract*. 2017;23(2):e12517. https://doi.org/10.1111/ijn.12517
16. Coyne BM, Kennedy C, Self A, Bullock L. A comprehensive approach to undergraduate nursing students' research experiences. *J Nurs Educ*. 2018;57(1):58–62. https://doi.org/10.3928/01484834-20180102-10
17. Henoch I, Ung EJ, Ozanne A, Falk H, Falk K, Sarenmalm EK, et al. Nursing students' experiences of involvement in clinical research: An exploratory study. *Nurse Educ Pract*. 2014;14(2):188–94. https://doi.org/10.1016/j.nepr.2013.08.010
18. Ünver S, Semerci R, Özkan ZK, Avcibaşi İ. Attitude of nursing students toward scientific research: A cross-sectional study in Turkey. *J Nurs Res*. 2018;26(5):356–61. https://doi.org/10.1097/jnr.0000000000000267
19. Chaturvedi D, Tak HK, Rawat VS. Assessment of attitude towards research among nursing students in Rajasthan, India. *Int J Adv Nurs Manag*. 2023;11(1):19–25.
20. Rholdon R, Templet T, Lemoine J, Hanna J, Craven C. An embedded EBP project: Effect on student nurses' practice, attitude, and knowledge. *Teach Learn Nurs*. 2021;16(2):110–5. https://doi.org/10.1016/j.teln.2020.09.003
21. Polit DF, Beck CT. *Essentials of Nursing Research: Appraising Evidence for Nursing Practice*. 9th ed. Philadelphia: Wolters Kluwer; 2020. DOI not available.
22. Faul F, Erdfelder E, Lang AG, Buchner A. G*Power 3: A flexible statistical power analysis program for the social, behavioral, and biomedical sciences. *Behav Res Methods*. 2007;39(2):175–91. https://doi.org/10.3758/BF03193146
23. IBM Corp. IBM SPSS Statistics for Windows, Version 26.0. Armonk, NY: IBM Corp; 2018.
24. Ayaz-Alkaya S, Terzi H. Effectiveness of scientific research education programme on the attitude of nursing students towards research methods course: A pre and post-implementation study. *Contemp Nurse*. 2024;60(1):1–9. https://doi.org/10.1080/10376178.2024.1871234
25. Erkin O, Toraman AU, Simsek H, Temel AB. Nursing students' attitudes toward research-development: Does taking research course make a difference. *Int J Caring Sci*. 2017;10(2):988–96.
26. Ross JG, Burrell SA, Mendes A, Heverly M. Pre-licensure nursing students' attitudes toward clinical research, education research, and pedagogical research participation in the United States. *Nurse Educ Today*. 2020;93:104522. https://doi.org/10.1016/j.nedt.2020.104522
27. Negru-Subtirica O, Pop EI, Crocetti E, Meeus W. Social comparison at school: Can GPA and personality mutually influence each other across time? *J Pers*. 2020;88(3):555–67. https://doi.org/10.1111/jopy.12506
28. Amoo TB, Gbadamosi O. Nursing and midwifery students' attitudes towards research: A descriptive study. *Asian J Nurs Educ Res*. 2021;11(3):375–80.
29. Oducado RM. Factors affecting nursing students' attitudes toward research: A survey in a Philippine higher education institution. *Indones Nurs Sci J*. 2021;11(1):1–8.

Figure Click here to access/download;Figure;TABLE.docx

Table 1. Sociodemographic and Research-related Characteristics among Students

Characteristic	Category	The original sample (N=62)	The final sample (N=56)	The original sample (N=62)	The final sample (N=56)
		**n (%)**	**n (%)**	**M (SD)**	**M (SD)**
Age	-			21.52 (0.86)	21.51 (0.85)
GPA	-			4.19 (0.47)	4.22 (0.47)
Sex	Male	23 (37.09%)	22 (39.28%)	-	-
	Female	39 (62.91%)	34 (60.72%)	-	-
Have you ever studied research courses/concepts before?	No	59 (95.16%)	54 (96.43%)	-	-
	Yes	3 (4.84%)	2 (3.57%)	-	-
Where did you study research courses/concepts?	First-year bachelor	2 (66.70%)	2 (100.0%)	-	-
	High school	1 (33.30%)	0 (0.0%)	-	-
Were you oriented to "research principles" before taking this research course?	No	53 (85.48%)	48 (85.71%)	-	-
	Yes	9 (14.52%)	8 (14.29%)	-	-
Where were you oriented to the "research principles" before taking this research course?	First-year bachelor	4 (44.44%)	4 (50.00%)	-	-
	High school	4 (44.44%)	3 (37.50%)	-	-
	Workshop	1 (11.12%)	1 (12.50%)	-	-
Have you used/applied research concepts before?	No	50 (80.64%)	46 (82.14%)	-	-
	Yes	12 (19.36%)	10 (17.86%)	-	-
Where did you use/apply research concepts?	Workshop	4 (33.32%)	4 (20.00%)	-	-
	Other courses	8 (66.68%)	6 (60.00%)	-	-

Note, N: Sample Size, M: mean, SD: standard deviation, %: percentage, n: frequency, GPA: Grade Point Average.

Table 2. Two-tailed paired samples t-test for the difference in students' attitudes before and after attending the nursing research course

Subcategory	Pre test M (SD)	Post test M (SD)	t	p	d
Overall Scale (22–88)	66.28 (8.43)	69.58 (9.91)	-2.48	0.01	55
Research Abilities (possible range 7–28)	20.53 (3.45)	21.96 (3.72)	-2.55	0.01	55
Using Research in Clinical Practice (possible range 7–28)	18.39 (4.26)	20.33 (4.65)	-2.82	.007	55
Usefulness of Research (possible range 4–16)	13.91 (1.65)	13.87 (2.06)	.137	.89	55
Personal Interest in Research (possible range 4–16)	13.44 (2.12)	13.41 (2.20)	.115	.90	55

Note, M: Mean, SD: Standard deviation, t: t test statistic, p: alpha level, d: Degrees of freedom.
