# Supplementary material for: Does attending a structured undergraduate nursing research course affect nursing students’ attitudes toward nursing research? A quasi-experimental study
Source: PLoS One. 2026 Jul 7;21(7):e0351538. doi: 10.1371/journal.pone.0351538 (PMC13340820; doi:10.1371/journal.pone.0351538)
Supplement: Attachment — Submitted filename: Response_to_reviewers_PONE-D-25-61156_auresp_2.docx [file pone.0351538.s007.docx]

# Response to Editor and Reviewers' Comments

# Manuscript Title: Does attending a structured undergraduate nursing research course affect nursing students’ attitudes toward nursing research? A quasi-experimental study

# PONE-D-25-61156

# We sincerely thank the Editor and all reviewers for their thorough evaluation of our manuscript and for the constructive comments provided. All comments have been carefully addressed, and the manuscript has been revised accordingly to enhance methodological rigour, clarity, and overall quality. For ease of review, all changes are highlighted in blue/ tracked in the manuscript. Below, we provide a detailed, point-by-point response.

| **Comment Number** | **Comment** | **Response / Action** | **Location (Section / Page)** |
| --- | --- | --- | --- |
| **General comments** | | | |
| 1. | Thank you for submitting the revised version of your manuscript. I have now completed my review and would like to acknowledge the effort you have invested in addressing the previous comments. | We sincerely thank the Editor and reviewers for the careful review and for acknowledging our efforts in revising the manuscript. | NA |
| 2. | The manuscript requires extensive language editing and refinement. | Thank you for this important comment. The manuscript has undergone thorough language editing and refinement to improve clarity, coherence, and overall readability. | Entire manuscript (Pages 1–end) |
| 3. | Please clearly disclose any use of AI tools in the preparation of this manuscript. There are sections of the text that appear to be AI-generated, and transparency in this regard is essential in accordance with publication ethics | Thank you for highlighting this important issue. We confirm that AI-assisted tools were used only for language editing and proofreading (e.g., improving grammar and clarity), and not for content generation, data analysis, or interpretation. A statement has now been added to the manuscript in accordance with the journal's requirements.  We carefully reviewed the sections that may have raised concern and ensured consistency in tone and academic style throughout the manuscript. | Declarations section (Page 24, Lines 466–469) |
| 4. | The manuscript must partially describe a technically sound piece of scientific research with data that supports the conclusions. Experiments must have been conducted rigorously, with appropriate controls, replication, and sample sizes. The conclusions must be drawn appropriately based on the data presented. | Thank you for this valuable comment. We have revised the manuscript to ensure that conclusions are fully supported by the data and aligned with the study design. Specifically, we clarified the absence of a control group, avoided causal language, reported effect sizes, and emphasised a cautious interpretation of the findings. Practical and research implications were also refined accordingly. | Results (Page 20, Lines 368–369)+(Page 14, Lines 250–262)  Strengths and limitations (Page 21-22, Lines 385–427)  Conclusion (Page 23, Lines 441–452) |
| 5. | The statistical analysis has been performed appropriately and rigorously | We thank the Editor for this positive evaluation. | NA. |
| 6. | The authors made all data underlying the findings in their manuscript fully available. | We confirm that all underlying data have been made fully available as supplementary files in accordance with journal requirements. | Data Availability Statement (Page 24, Lines 256–259) |
| 7. | The manuscript was presented in an intelligible fashion and written in standard English. | We appreciate this positive feedback. The manuscript has been further refined for clarity and readability. | NA |
| Reviewer #1 | | | |
| 1. | Please rewrite the title in statement form as a short title. It would be better to state it as "Impact," as the findings are merely associated but have not established a temporal relationship. Also, add the population. | Thank you for this important suggestion. We revised the title to avoid causal language and reflect the quasi-experimental design. Terms such as “impact” were replaced with “association,” and the study population was clarified. The short title was also revised accordingly. | Title page (Page 1, Line 5) |
| 2. | In the result section, n=56, so the findings will be for 56 only, not for 62.  The correlation can be better shown with plots than with text results. | Thank you for this observation. We clarified that all inferential analyses were conducted on the final sample (n = 56) after excluding participants with incomplete post-test data.  We have also included graphical representations (scatter plots) to better illustrate the correlations between variables, improving clarity and interpretation. | Results section (Page 11-12, Lines 232–243)+(Page 8, Lines 150-151)  Results section (Figure 1- attached to the manuscript) |
| 3. | Please mention that the 14-week intervention has been measured only after 5 months, and this causes problems in presenting your results, just because of your intervention. The posttest findings are contaminated. It would have been much better if you have included other standarization of mesurement of variables such as research critique practice, clinical skills measurement techniques etc. You have mentioned as you have standarized but also mention in methodlogy section about how did you standarize? You have highlighted such teaching learning methodologies in literature gaps but not included in your study as a tool. You can keep it as a limitations of your study and further recommendations for other researchers. | Thank you for this insightful comment. We clarified the study timeline, noting that although the intervention lasted 14 weeks, the overall study period corresponded to the academic semester (~4 months). We also expanded the Intervention section to describe standardisation procedures (e.g., unified syllabus, teaching materials, and assessment methods). Additionally, we acknowledged as a limitation that only attitudes were measured, without including other outcomes such as research skills or clinical competencies, and recommended these for future research. | Methods (Page 11, Lines 214–217) + (Page 9, Lines 182–188)  Limitations (Page 22, Lines 423–428) |
| Reviewer #2: | | | |
| 1. | All the comments have been addressed. | We thank the reviewer for their positive evaluation and for acknowledging that all comments have been satisfactorily addressed. | NA |
| 2. | It would have been better to mention whether the 14 weeks course included any hands-on training sessions. Were the students involved in any actual research project during the course? | Thank you for this valuable suggestion. We have clarified that the course included practical, hands-on learning activities such as literature searching, critical appraisal, and participation in small-scale research-related tasks. We also specified the extent of student involvement in research-related activities during the course to improve transparency of the intervention. | Intervention section (Page 8-9, Lines 153–188) |
