# Supplementary material for: Does attending a structured undergraduate nursing research course affect nursing students’ attitudes toward nursing research? A quasi-experimental study
Source: PLoS One. 2026 Jul 7;21(7):e0351538. doi: 10.1371/journal.pone.0351538 (PMC13340820; doi:10.1371/journal.pone.0351538)
Supplement: Attachment — Submitted filename: PONE-D-25-61156_reviewer_comment_RD_17DEC2025.docx [file pone.0351538.s004.docx]

**Manuscript Number PONE-D-25-61156**

**Understanding Nursing Students’ Attitudes Toward Research A Quasi-experimental Study**

- This study tests an important, but unproven hypothesis: whether the investment in a required research course leads to positive attitude changes in the classroom.

**Title:** (The current title is informative; however, consider whether a more active title would be more appropriate, for example, "Impact of a Research-Focused Intervention on Nursing Students' Attitudes...")

**Introduction**:

- The introduction follows a good funnel structure.
- The first paragraph lists studies reporting both positive and negative attitudes. It is recommended, however, to begin with a statement that directly links research to patient outcomes. Establishing the importance of nursing research and evidence-based practice (EBP) in the healthcare sector
  - *Example: "Evidence-based practice (EBP), supported by rigorous nursing research, provides high-quality, safe patient care and advances the nursing profession globally."*
- The transition about cultural difference is a bit abrupt {line 84}. Integrate the context factors more smoothly
- In the second paragraph [line 86], effective strategies are listed. Provide a critical link to your study. You need to transition from this list of successful interventions to why your study remains important.
- The paragraph concerning faculty and course methods (beginning on line 98) seems disconnected. Reframe the final two paragraphs into one cohesive argument emphasizing the importance of pedagogical evidence.
- The final gap statement (line 104). "Additional research studies are required to determine the appropriate learning and teaching strategies...". Be sure to make your statement specific to the design of your study. The gap is significant and generalized. It should be specific to the intervention and location (KSA) you used.
- Study Aim. Make use of stronger, more active language (for an intervention study).
  - *Suggestion: "This study aimed to examine how a structured, credit-bearing undergraduate nursing research course influenced Saudi nursing students' attitudes toward nursing research, utilizing a pre-test/post-test quasi-experimental design."*

**Methods**:

- It should be organized more clearly, characterized by a distinction between initial enrollment and final analysis.
- Specify the exact duration, the tools used, and the ethics approval number.

**Results:**

- Clarify the Sample Flow: Identify the final size of the analytic sample. Consider using a CONSORT/Flow Diagram concept (or explain the reason for exclusion) as soon as possible.
- Only the characteristics of the N=56 students used for the analysis are relevant to the results. To avoid redundancy, delete the initial N=62 description. In table 1, keep the data regarding the final sample (n=56).
- In table 1: Under the characteristics column, rephrase the question in shorter terms, for example: Were you oriented to research principles before taking this research course?” to [prior research course] and in the same way for the other questions.
- Is it the same six students who withdrew who did not complete the post-test?
- It is recommended that p-values be standardized by using a leading zero (e.g., p=0.01). Report results that are statistically significant up to two or three decimal places
- The report contains the mean difference and confidence interval (CI), which is excellent. For the paired t-test, however, a standardized effect size must be used (e.g., Cohen's d)
- Lines 210 to 224 of the report explain the overall significant finding, the two significant subscales, and the non-significant subscales, before repeating the detail for the two significant subscales. We recommend not repeating the detailed description.
- There are four subscales on your questionnaire: Research Abilities, Using Research in Clinical Practice, Personal Interest in Research, and Usefulness of Research. Please ensure that these exact and consistent names are used throughout the entire document.
- In line 228-233, the reporting correlation is r=-0.31, p=0.02. Although this is correct, it does not provide the full context. Be sure to use correct notation and interpretation: Identify the full test (e.g., Pearson's r), the correlation coefficient (r), the degrees of freedom (N-2), and the p-value.
- Interpreting GPA correlations. It should be noted that the finding is a significant inverse correlation (r = -0.33, p = 0.01). It is a very interesting, non-obvious finding that should be emphasized clearly.
  - *Example: "A significant inverse correlation was observed between GPA and total pre-test attitudes (r(54)=-0.33, p=0.01), suggesting that students with higher academic achievement reported lower attitudes toward research."*
- The post-test correlation is missing. Only pre-test correlations are included in the report. Were these characteristics correlated with post-test scores or did they affect scores?

**Discussion**

- The discussion should be even more structured, critical, and focused on linking the specific components of your intervention to the observed attitudinal change.
- While the paragraphs flow well, the key components are mixed throughout.
- An overview of the key findings. This is covered in the first sentence, but it is weak. Prior to comparison with literature, a dedicated, clear summary statement is required.
- Include a paragraph dedicated to Interpretation of Specific Sub-scales, clearly linking the improvement to specific intervention strategies.

Linking Intervention to Results:

- Line 282-283: "The research course in the present study involved different strategies, such as mini-projects, critiquing articles, in-class teaching, and discussions". A discussion section would be better suited for a more specific description. It is essential to indicate which specific strategy led to the improvement in the significant domains.
- Paragraph starting at Line 251**:** "The findings contradict... Australia, which revealed no significant change... instruction was online, which differed from the method... This suggests a potential benefit of using interactive and in-class instruction...". The point is well taken, but it should be strengthened. Describe the difference in terms of a hypothesis for future research.
  - Suggestion: "These conflicting outcomes underscore the importance of pedagogical modality; the non-significant findings in the Australian study, using online instruction (11), versus our face-to-face, interactive approach, suggest that direct student-instructor interaction may be a prerequisite to attitude change."
- In my opinion, the interpretation regarding GPA (academic pressure) is compelling. Be sure to specify that these are correlations with the pre-test score and that future studies should examine whether high-GPA students also show a lower rate of improvement (i.e., less course effect).
