# Supplementary material for: Does attending a structured undergraduate nursing research course affect nursing students’ attitudes toward nursing research? A quasi-experimental study
Source: PLoS One. 2026 Jul 7;21(7):e0351538. doi: 10.1371/journal.pone.0351538 (PMC13340820; doi:10.1371/journal.pone.0351538)
Supplement: Attachment — Submitted filename: Response to reviewers PONE-D-25-61156.docx [file pone.0351538.s006.docx]

# We sincerely thank the Editor and all reviewers for their thorough evaluation of our manuscript and for the constructive comments provided. All comments were carefully addressed, and the manuscript was revised accordingly to improve methodological rigour, clarity, and overall quality. All changes made in response to the reviewers’ comments are highlighted in light blue in the revised manuscript. Below, we provide a detailed, point-by-point response.

# Response to Editor and Reviewers Comments

| **Manuscript Title: Understanding Nursing Students’ Attitudes Toward Research A Quasi-experimental Study**  **PONE-D-25-61156** | | | |
| --- | --- | --- | --- |
| **Comment Number** | **Comment** | **Response / Action** | **Location (Section / Page)** |
| **Editor** | | | |
| 1. | Please ensure that your manuscript meets PLOS ONE's style requirements, including those for file naming | Thank you for this comment. The manuscript has been carefully revised to ensure full compliance with PLOS ONE’s formatting and style requirements. File naming conventions, manuscript structure, headings, and overall presentation were reviewed and adjusted accordingly. | Entire manuscript (Pages 1–29) |
| 2. | Please include your tables as part of your main manuscript and remove the individual files. Please note that supplementary tables (should remain/ be uploaded) as separate "supporting information" files. | Thank you for the clarification. All tables have now been fully embedded within the main manuscript at their appropriate locations and are no longer submitted as separate supporting information files. | Results section – Tables 1 and 2 (Pages 13–14)  + Supplementary File (s1) |
| 3. | We note that the grant information you provided in the ‘Funding Information’ and ‘Financial Disclosure’ sections do not match.  When you resubmit, please ensure that you provide the correct grant numbers for the awards you received for your study in the ‘Funding Information’ section. | The funding information has been reviewed for consistency. All funding details have been standardised to ensure accurate reporting of the grant number and funding source. | Funding section (Page 23) ?????  ?? |
| 4. | . Thank you for stating the financial disclosure.  Please state what role the funders took in the study. If the funders had no role, please state: "The funders had no role in study design, data collection and analysis, decision to publish, or preparation of the manuscript." If this statement is not correct you must amend it as needed. Please include this amended Role of Funder statement in your cover letter; we will change the online submission form on your behalf. | A clear funder role statement has been added, specifying that the funders had no role in the study design, data collection and analysis, decision to publish, or preparation of the manuscript. | Funding section (Page 23) ?????  ??  + Cover letter ? |
| 5. | Thank you for stating the following in the Acknowledgments Section of your manuscript: “The researchers gratefully acknowledge Princess Nourah bint Abdulrahman University Researchers Supporting Project number (PNURSP2025R444), Princess Nourah bint Abdulrahman University, Riyadh, Saudi Arabia.”  We note that you have provided funding information that is not currently declared in your Funding Statement. However, funding information should not appear in the Acknowledgments section or other areas of your manuscript. We will only publish funding information present in the Funding Statement section of the online submission form. Please remove any funding-related text from the manuscript and let us know how you would like to update your Funding Statement. Currently, your Funding Statement reads as follows: “The research study received funding from Princess Nourah bint Abdulrahman University Researchers Supporting Project number (PNURSP2025R444), Princess Nourah bint Abdulrahman University, Riyadh, Saudi Arabia. “  Please include your amended statements within your cover letter; we will change the online submission form on your behalf. | All funding-related text has been removed from the Acknowledgments section. Funding information is now reported exclusively in the Funding section, in accordance with PLOS ONE guidelines. | Acknowledgments section (Page 24); Funding section (Page 23) |
| 6. | In the online submission form, you indicated that “The data that support the findings of this study are available on request from the corresponding author.”  All PLOS journals now require all data underlying the findings described in their manuscript to be freely available to other researchers, either 1. In a public repository, 2. Within the manuscript itself, or 3. Uploaded as supplementary information.  This policy applies to all data except where public deposition would breach compliance with the protocol approved by your research ethics board. If your data cannot be made publicly available for ethical or legal reasons (e.g., public availability would compromise patient privacy), please explain your reasons on resubmission and your exemption request will be escalated for approval. | The Data Availability Statement has been updated. The dataset underlying the findings has been uploaded as Supplementary Files S1 + S2 and are freely available. | Declarations – Data Availability Statement (Page 22); Supplementary File S1 +S2 |
| 7. | PLOS requires an ORCID iD for the corresponding author in Editorial Manager on papers submitted after December 6th, 2016. Please ensure that you have an ORCID iD and that it is validated in Editorial Manager. To do this, go to ‘Update my Information’ (in the upper left-hand corner of the main menu), and click on the Fetch/Validate link next to the ORCID field. This will take you to the ORCID site and allow you to create a new iD or authenticate a pre-existing iD in Editorial Manager. | The corresponding author’s ORCID iD has been provided and validated in the Editorial Manager system. | Title page (Page 1) |
| 8. | Your ethics statement should only appear in the Methods section of your manuscript. If your ethics statement is written in any section besides the Methods, please move it to the Methods section and delete it from any other section. Please ensure that your ethics statement is included in your manuscript, as the ethics statement entered into the online submission form will not be published alongside your manuscript. | The ethics statement has been placed exclusively in the Methods section and removed from all other parts of the manuscript. | Ethics Statement subsection (Page 10) |
| 9. | If the reviewer comments include a recommendation to cite specific previously published works, please review and evaluate these publications to determine whether they are relevant and should be cited. There is no requirement to cite these works unless the editor has indicated otherwise. | No additional published words were recommended. | - |
| 10. | We appreciate your submission to PLOS ONE. The reviewers have provided detailed feedback and highlighted a number of issues that require your attention. We kindly ask that you address these comments carefully in your revision. | Thank you. All reviewer and editor comments were carefully addressed, and the manuscript was revised accordingly to improve clarity, methodological rigor, and compliance with PLOS ONE requirements. | - |
| **Reviewer 1** | | | |
| 1. | Thank you so much for writing manuscript in nursing student's attitude towards nursing research. The topic is very much evolving. However, go through the review attached in the pdf to make a robust manuscript. | Thank you. The manuscript was carefully revised in accordance with all reviewer and editor recommendations, and all identified issues were fully addressed in the revised version. | - |
| 2. | Please write the introduction section with more elaboration of your variables of the study first and then digging deep into the need of nursing student's attitude in research in a funneling way. You have mentioned the status globally but not reaching into your country and at the local level of your study area. Please rewrite the introduction section. | The Introduction has been substantially revised to adopt a clearer funnel structure, beginning with the global importance of nursing research and evidence-based practice, followed by regional and Saudi-specific context, and concluding with the local research gap addressed by this study. | Introduction (Pages 4 - 6) |
| 3. | Please explain the sample size calculation in detail. You can add supplementary file or include it in the manuscript itself. What is the reference for augmenting the sample size by 20%? | The sample size calculation has been expanded, including justification for inflating the sample size to account for potential attrition, supported by methodological literature. | Methods – Samples and Sampling Technique (Pages 7–8) |
| 4. | Please keep the instrument of 14 weeks course in supplementary file for clarification. This should be well measurable. | A detailed description of the course content has been provided as Supplementary File S1. | Methods – Intervention (Page 9); & Supplementary File S1 |
| 5. | Please explain the data collection procedure in detail. Explaining if any sample attrition, reason behind the gap of 5 months for posttest. Since the course is of 14 weeks how long the period was there before post test? | The data collection process has been clarified, including reasons for attrition, timing of the post-test, and alignment with the academic semester. | Methods – Data Collection (Page 10+11) |
| 6. | Please add more about data cleaning, editing, coding . Explain the nature of the data , distribution of the data in terms of normality etc. | A detailed description of data screening, coding, and normality assessment has been added to the Data Analysis section. | Methods – Data Analysis (Page 11) |
| 7. | The result about the attitude of the nursing students towards nursing research was measured and significant findings are explained based on the intervention as a 14 weeks course , but how do you define whether the findings are just because of the interventions you carried out or because of other confounders as findings from pre intervention knowledge and research utilization you have mentioned in the study.  Please clarify the results.  what was done to the students who already had knowledge and utilized the research ? were they still included in the study? Your findings are not clear based on the study design you have adopted. Please clarify it. | The Methodes, Results, and Discussion sections were revised to acknowledge potential confounding factors and to interpret findings as associations rather than causal effects. | Sampling technique/ page 7.  Results (Table 1); Discussion (Pages 16-17, 20) |
| 8. | The limitations of the study are well explained. However, the measures could have been taken to minimize such limitations in this study itself. Please justify it . | The limitations section now explicitly describes measures taken to minimise bias, including standardisation of teaching and synchronised data collection. | Discussion – Strengths and Limitations (Pages 20-21) |
| 9. | Conclusion: please make correction as "finding" | The wording in the Conclusion has been corrected. | Conclusion (Page 22) |
| **Reviewer 2 comments** | | | |
| 1. | 1.The research objective is to assess the attitude towards research, but the content seem to divert towards teaching and learning methods. | The manuscript has been revised to ensure teaching strategies are discussed strictly as components of the intervention influencing attitudes, maintaining alignment with the stated objective. | Introduction (Pages 4–6);  Discussion (Pages 16–17) |
| 2. | 2. Did all of the students attend complete 14 weeks course?? were none of them absent?? | Attendance and completion of the 14-week course were clarified, and attrition was explicitly reported and accounted for analytically. | Methods – Data Collection (Page 10);  Results – Sample Description (Page 11) |
| **Reviewer 3 comments** | | | |
| 1. | This study tests an important, but unproven hypothesis: whether the investment in a required research course leads to positive attitude changes in the classroom. | We appreciate this observation, and the Introduction and Discussion were revised to frame the study as an examination of associations between course participation and attitude changes, with cautious interpretation of findings. | Results (Page 11);  Discussion (Page 15) |
| 2. | Title: (The current title is informative; however, consider whether a more active title would be more appropriate, for example, "Impact of a Research-Focused Intervention on Nursing Students' Attitudes...") | The title was revised to adopt a more active and intervention-focused phrasing. | Title page (Page 1) |
| 3. | The introduction follows a good funnel structure.  The first paragraph lists studies reporting both positive and negative attitudes. It is recommended, however, to begin with a statement that directly links research to patient outcomes. Establishing the importance of nursing research and evidence-based practice (EBP) in the healthcare sector o Example: "Evidence-based practice (EBP), supported by rigorous nursing research, provides high-quality, safe patient care and advances the nursing profession globally." | The opening paragraph of the Introduction was revised to explicitly link nursing research and evidence-based practice to patient outcomes. | Introduction (Page 4) |
| 4. | The transition about cultural difference is a bit abrupt {line 84}. Integrate the context factors more smoothly | Transitions was revised to integrate cultural and contextual factors more coherently. | Introduction (Page 5) |
| 5. | In the second paragraph [line 86], effective strategies are listed. Provide a critical link to your study. You need to transition from this list of successful interventions to why your study remains important. | The Introduction was revised to explicitly connect previously reported effective research-teaching strategies to the remaining research gap, clearly justifying the need for the present structured, context-specific intervention. | Introduction (Pages 4-5) |
| 6. | The paragraph concerning faculty and course methods (beginning on line 98) seems disconnected. Reframe the final two paragraphs into one cohesive argument emphasizing the importance of pedagogical evidence. | The final two paragraphs of the were merged and reframed into a single cohesive argument emphasizing the importance of pedagogical evidence and faculty-led instructional approaches in shaping students’ research attitudes. | Introduction (Pages 5-6) |
| 7. | The final gap statement (line 104). "Additional research studies are required to determine the appropriate learning and teaching strategies...". Be sure to make your statement specific to the design of your study. The gap is significant and generalized. It should be specific to the intervention and location (KSA) you used. | The research gap and study aim were rewritten using stronger, intervention-specific language relevant to the Saudi context. | Introduction – (Page 6) |
| 8. | Study Aim. Make use of stronger, more active language (for an intervention study). o Suggestion: "This study aimed to examine how a structured, credit-bearing undergraduate nursing research course influenced Saudi nursing students' attitudes toward nursing research, utilizing a pre-test/post-test quasi-experimental design." | The Study Aim was revised using stronger, intervention-focused language, explicitly describing the structured, credit-bearing research course and the quasi-experimental pre-test/post-test design. | Introduction – Study Aim (Page 6) |
| 9. | Methods:  It should be organized more clearly, characterized by a distinction between initial enrollment and final analysis. | The Methods section was reorganized to clearly distinguish initial enrollment, eligibility criteria, attrition, and the final analytic sample. | Methods – Samples and Sampling Technique & Data Collection (Pages 7-8 , 10-11) |
| 10. | Specify the exact duration, the tools used, and the ethics approval number. | The exact course duration (14 weeks), data collection instrument, and ethics approval number were explicitly specified in the Methods section. | Methods – Study Design; Instrument; Ethics Statement (Pages 9–10) |
| 11. | Results:Clarify the Sample Flow: Identify the final size of the analytic sample. Consider using a CONSORT/Flow Diagram concept (or explain the reason for exclusion) as soon as possible. | Sample flow was clarified at the beginning of the Results section by reporting initial enrollment, reasons for exclusion, and the final analytic sample size (n = 56). | Results – Sample Description (Page 11) |
| 12. | Only the characteristics of the N=56 students used for the analysis are relevant to the results. To avoid redundancy, delete the initial N=62 description. In table 1, keep the data regarding the final sample (n=56). | Sample flow has been clarified in the Results section, and both samples were retained in Table 1 to demonstrate comparability. | Results – (Pages 11, 13) |
| 13. | In table 1: Under the characteristics column, rephrase the question in shorter terms, for example: Were you oriented to research principles before taking this research course?” to [prior research course] and in the same way for the other questions. | The wording of characteristics in Table 1 was shortened and standardized to improve clarity and readability. | Results – Table 1 (Page 13) |
| 14. | Is it the same six students who withdrew who did not complete the post-test? | Yes. The same six students withdrew and did not complete the post-test; this has been explicitly clarified in the Methods and Results sections. | Data Collection (Page 10); Results – Sample Description (Page 11) |
| 15. | It is recommended that p-values be standardized by using a leading zero (e.g., p=0.01). Report results that are statistically significant up to two or three decimal places | P-values were standardised using leading zeros and consistent decimal formatting throughout. | Results – (Pages 11-15) |
| 16. | The report contains the mean difference and confidence interval (CI), which is excellent. For the paired t-test, however, a standardized effect size must be used (e.g., Cohen's d) | Cohen’s d effect sizes were added, reported, and interpreted in both the Results and Discussion sections. | Results – Table 2 (Page 14); Discussion (Page 17) |
| 17. | Lines 210 to 224 of the report explain the overall significant finding, the two significant subscales, and the non-significant subscales, before repeating the detail for the two significant subscales. We recommend not repeating the detailed description. | Thank your for this comment. We deleted the redundant information. | Results – (Page 12) |
| 18. | There are four subscales on your questionnaire: Research Abilities, Using Research in Clinical Practice, Personal Interest in Research, and Usefulness of Research. Please ensure that these exact and consistent names are used throughout the entire document. | The four subscales were standardized and used consistently throughout the manuscript. | Entire manuscript (Pages 1–29) |
| 19. | In line 228-233, the reporting correlation is r=-0.31, p=0.02. Although this is correct, it does not provide the full context. Be sure to use correct notation and interpretation: Identify the full test (e.g., Pearson's r), the correlation coefficient (r), the degrees of freedom (N-2), and the p-value. | The correlation analysis was revised to include full statistical reporting, specifying Pearson’s correlation coefficient, degrees of freedom (r(df)), and p-value, along with appropriate interpretation. | Results – (Page 15) |
| 20. | Interpreting GPA correlations. It should be noted that the finding is a significant inverse correlation (r = -0.33, p = 0.01). It is a very interesting, non-obvious finding that should be emphasized clearly. o Example: "A significant inverse correlation was observed between GPA and total pre-test attitudes (r(54)=-0.33, p=0.01), suggesting that students with higher academic achievement reported lower attitudes toward research."  The post-test correlation is missing. Only pre-test correlations are included in the report. Were these characteristics correlated with post-test scores or did they affect scores? | The correlation analysis has been clarified, explicitly distinguishing pre-test from post-test associations and identifying findings as exploratory. | Results – (Page 12)  Discussion – (page 19) |
| 21. | The discussion should be even more structured, critical, and focused on linking the specific components of your intervention to the observed attitudinal change.  While the paragraphs flow well, the key components are mixed throughout. | The Discussion section was reorganized to improve structure and critical focus, with clearer separation between key findings, interpretation, and comparison with existing literature. | Discussion – (page 15-21) |
| 22. | An overview of the key findings. This is covered in the first sentence, but it is weak. Prior to comparison with literature, a dedicated, clear summary statement is required. | A concise summary paragraph outlining the key findings was added prior to comparison with previous studies. | Discussion – (pages 15-21) |
| 23. | Include a paragraph dedicated to Interpretation of Specific Sub-scales, clearly linking the improvement to specific intervention strategies. Linking Intervention to Results: | A dedicated subsection was added to interpret changes in subscales, explicitly linking improvements to specific course components and learning activities. | Discussion – (page 17-18) |
| 24. | Line 282-283: "The research course in the present study involved different strategies, such as mini-projects, critiquing articles, in-class teaching, and discussions". A discussion section would be better suited for a more specific description. It is essential to indicate which specific strategy led to the improvement in the significant domains. | The Discussion was revised to explicitly link mini-projects, article critiques, and applied classroom activities to improvements in research abilities and research use in clinical practice. | Discussion – (page 17-18) |
| 25. | Paragraph starting at Line 251: "The findings contradict... Australia, which revealed no significant change... instruction was online, which differed from the method... This suggests a potential benefit of using interactive and in-class instruction...". The point is well taken, but it should be strengthened. Describe the difference in terms of a hypothesis for future research. o Suggestion: "These conflicting outcomes underscore the importance of pedagogical modality; the non-significant findings in the Australian study, using online instruction (11), versus our face-to-face, interactive approach, suggest that direct student-instructor interaction may be a prerequisite to attitude change." | This section was strengthened by reframing the comparison as a testable hypothesis, emphasizing pedagogical modality and suggesting that face-to-face interaction may be critical for attitude change. | Discussion – (page 16) |
| 26. | In my opinion, the interpretation regarding GPA (academic pressure) is compelling. Be sure to specify that these are correlations with the pre-test score and that future studies should examine whether high-GPA students also show a lower rate of improvement (i.e., less course effect). | The Discussion now explicitly states that GPA correlations relate to pre-test attitudes only and highlights the need for future studies to examine whether high-GPA students demonstrate differential intervention effects. | Discussion (Page 19) |
| **Reviewer 4** | | | |
| 1. | Limitations of the Study Design and Causal Interpretation The study employs a single-group pretest–posttest design without a control group. While this design is acknowledged in the limitations section, the manuscript occasionally uses language that implies a causal effect of the course on students’ attitudes (e.g., “effect,” “impact,” “improved by attending the course”). Given the absence of a comparison group, alternative explanations such as maturation, concurrent coursework, or increased academic exposure over the semester cannot be ruled out. | Causal language has been revised throughout the manuscript, replacing terms with more cautious wording (e.g., “associated with”). | Entire manuscript (Pages 1–29) |
| 2. | Recommendations: Revise wording throughout the Abstract, Discussion, and Conclusion to avoid causal implications. Use more cautious language such as “was associated with,” “was followed by,” or “coincided with.” | Causal language has been revised throughout the manuscript, replacing terms with more cautious wording (e.g., “associated with”). | Entire manuscript (Pages 1–29) |
| 3. | Expand the discussion of potential confounding factors that could have contributed to changes in attitudes independent of the course itself. | The Discussion and Limitations sections were expanded to explicitly address maturation, concurrent coursework, and other confounders. | Discussion – (page 19)  Discussion – Strengths and Limitations (Pages 20-21) |
| 4. | 2. Insufficient Detail on the Educational Intervention Although the manuscript outlines the general content of the nursing research course, the description remains too broad to support reproducibility or meaningful comparison with other educational interventions. Recommendations: Clarify which learning activities were mandatory for all students (e.g., mini-projects, article critiques, oral presentations). Specify how student performance was assessed and whether research-related activities contributed to course grades. Consider providing a concise week-by-week overview or course framework, possibly as supplementary material. | Mandatory activities, assessment methods, and course structure were clarified, with a detailed framework provided as supplementary material. | Methods – Intervention (Pages 9); Supplementary File S1 |
| 5. | 3. Statistical Analysis and Interpretation of Effect Size The authors appropriately report paired t-test results and Cohen’s d. However, the interpretation of the effect size is limited. The reported Cohen’s d of 0.34 indicates a small to modest effect, which warrants a more nuanced discussion regarding its educational and practical significance. Recommendations: Elaborate on what a small-to-moderate effect size represents in the context of educational interventions. Discuss whether the observed changes are likely to be meaningful in real-world educational or clinical settings. Consider reporting and interpreting change scores or confidence intervals more explicitly to strengthen the analytical narrative. | The educational and practical significance of the small-to-moderate effect size and confidence intervale were explicitly discussed. | Results – Table 2 (Page 14); Discussion (Page 17) |
| 6. | 4. Interpretation of the Negative Association Between GPA and Research Attitudes The finding that higher GPA is negatively correlated with attitudes toward research is potentially interesting, but the explanation provided is largely speculative. Recommendations: Clearly distinguish empirical findings from interpretative hypotheses. Acknowledge this result as exploratory rather than definitive. Consider alternative explanations, such as academic pressure, assessment structure, or sampling characteristics, and explicitly state that further research is needed to clarify this relationship. | Interpretations were clearly labelled as exploratory hypotheses, and alternative explanations were acknowledged. | Discussion – (page 19-20) |
| 7. | There is an inconsistency in the reported SPSS version (version 23 in the Methods section versus version 26 in the references); please standardize. | The SPSS version was standardized throughout the manuscript. | Methods – Data Analysis (Page 11); References |
| 8. | In Table 2, the column labeled “d” refers to degrees of freedom rather than effect size, which may confuse readers. Please revise the label or clarify in the table note. | Table 2 was revised to clearly distinguish degrees of freedom (df) from effect size. | Results – Table 2 (Page 14) |
| 9. | Minor grammatical and stylistic inconsistencies are present，British vs. American spelling; a careful language edit is recommended. | The manuscript was carefully edited to ensure consistent language usage and spelling throughout. | Entire manuscript (Pages 1–29) |
| 10. | The Introduction could be slightly condensed to improve focus on the specific research gap addressed by the study. | The Introduction was condensed to improve focus on the specific research gap and study rationale. | Introduction (Pages 4–6) |
