# Supplementary material for: Does attending a structured undergraduate nursing research course affect nursing students’ attitudes toward nursing research? A quasi-experimental study
Source: PLoS One. 2026 Jul 7;21(7):e0351538. doi: 10.1371/journal.pone.0351538 (PMC13340820; doi:10.1371/journal.pone.0351538)
Supplement: Peer review history

# Peer review: Decision Letter 0

**DOI:** 10.1371/journal.pone.0351538.r001

**Authors:** Ahmed El-Sayed

4 Jan 2026

PONE-D-25-61156

Understanding Nursing Students’ Attitudes Toward Research A Quasi-experimental Study

PLOS One

Dear Dr. AL-Mugheed,

Thank you for submitting your manuscript to PLOS ONE. After careful consideration, we feel that it has merit but does not fully meet PLOS ONE’s publication criteria as it currently stands. Therefore, we invite you to submit a revised version of the manuscript that addresses the points raised during the review process.

Please submit your revised manuscript by Feb 18 2026 11:59PM. If you will need more time than this to complete your revisions, please reply to this message or contact the journal office at plosone@plos.org. When you're ready to submit your revision, log on to https://www.editorialmanager.com/pone/ and select the 'Submissions Needing Revision' folder to locate your manuscript file.

Please include the following items when submitting your revised manuscript:

- A letter that responds to each point raised by the academic editor and reviewer(s). You should upload this letter as a separate file labeled 'Response to Reviewers'.
- A marked-up copy of your manuscript that highlights changes made to the original version. You should upload this as a separate file labeled 'Revised Manuscript with Track Changes'.
- An unmarked version of your revised paper without tracked changes. You should upload this as a separate file labeled 'Manuscript'.

If you would like to make changes to your financial disclosure, please include your updated statement in your cover letter. Guidelines for resubmitting your figure files are available below the reviewer comments at the end of this letter.

If applicable, we recommend that you deposit your laboratory protocols in protocols.io to enhance the reproducibility of your results. Protocols.io assigns your protocol its own identifier (DOI) so that it can be cited independently in the future. For instructions see: https://journals.plos.org/plosone/s/submission-guidelines#loc-laboratory-protocols. Additionally, PLOS ONE offers an option for publishing peer-reviewed Lab Protocol articles, which describe protocols hosted on protocols.io. Read more information on sharing protocols at https://plos.org/protocols?utm_medium=editorial-email&utm_source=authorletters&utm_campaign=protocols.

We look forward to receiving your revised manuscript.

Kind regards,

Ahmed Abdelwahab Ibrahim El-Sayed

Academic Editor

PLOS One

Journal Requirements:

When submitting your revision, we need you to address these additional requirements.

1. Please ensure that your manuscript meets PLOS ONE's style requirements, including those for file naming. The PLOS ONE style templates can be found at

https://journals.plos.org/plosone/s/file?id=wjVg/PLOSOne_formatting_sample_main_body.pdf and

https://journals.plos.org/plosone/s/file?id=ba62/PLOSOne_formatting_sample_title_authors_affiliations.pdf

2. Please include your tables as part of your main manuscript and remove the individual files. Please note that supplementary tables (should remain/ be uploaded) as separate "supporting information" files.

3. We note that the grant information you provided in the ‘Funding Information’ and ‘Financial Disclosure’ sections do not match.

When you resubmit, please ensure that you provide the correct grant numbers for the awards you received for your study in the ‘Funding Information’ section.

4. Thank you for stating the following financial disclosure:

“The research study received funding from Princess Nourah bint Abdulrahman University Researchers Supporting Project number (PNURSP2025R444), Princess Nourah bint Abdulrahman University, Riyadh, Saudi Arabia. “

Please state what role the funders took in the study. If the funders had no role, please state: "The funders had no role in study design, data collection and analysis, decision to publish, or preparation of the manuscript."

If this statement is not correct you must amend it as needed.

Please include this amended Role of Funder statement in your cover letter; we will change the online submission form on your behalf.

5. Thank you for stating the following in the Acknowledgments Section of your manuscript:

“The researchers gratefully acknowledge Princess Nourah bint Abdulrahman University Researchers Supporting Project number (PNURSP2025R444), Princess Nourah bint Abdulrahman University, Riyadh, Saudi Arabia.”

We note that you have provided funding information that is not currently declared in your Funding Statement. However, funding information should not appear in the Acknowledgments section or other areas of your manuscript. We will only publish funding information present in the Funding Statement section of the online submission form.

Please remove any funding-related text from the manuscript and let us know how you would like to update your Funding Statement. Currently, your Funding Statement reads as follows:

“The research study received funding from Princess Nourah bint Abdulrahman University Researchers Supporting Project number (PNURSP2025R444), Princess Nourah bint Abdulrahman University, Riyadh, Saudi Arabia. “

Please include your amended statements within your cover letter; we will change the online submission form on your behalf.

6. In the online submission form, you indicated that “The data that support the findings of this study are available on request from the corresponding author.”

All PLOS journals now require all data underlying the findings described in their manuscript to be freely available to other researchers, either 1. In a public repository, 2. Within the manuscript itself, or 3. Uploaded as supplementary information.

This policy applies to all data except where public deposition would breach compliance with the protocol approved by your research ethics board. If your data cannot be made publicly available for ethical or legal reasons (e.g., public availability would compromise patient privacy), please explain your reasons on resubmission and your exemption request will be escalated for approval.

7. PLOS requires an ORCID iD for the corresponding author in Editorial Manager on papers submitted after December 6th, 2016. Please ensure that you have an ORCID iD and that it is validated in Editorial Manager. To do this, go to ‘Update my Information’ (in the upper left-hand corner of the main menu), and click on the Fetch/Validate link next to the ORCID field. This will take you to the ORCID site and allow you to create a new iD or authenticate a pre-existing iD in Editorial Manager.

8. Your ethics statement should only appear in the Methods section of your manuscript. If your ethics statement is written in any section besides the Methods, please move it to the Methods section and delete it from any other section. Please ensure that your ethics statement is included in your manuscript, as the ethics statement entered into the online submission form will not be published alongside your manuscript.

9. If the reviewer comments include a recommendation to cite specific previously published works, please review and evaluate these publications to determine whether they are relevant and should be cited. There is no requirement to cite these works unless the editor has indicated otherwise.

**Additional Editor Comments**

Dear Authors,

We appreciate your submission to PLOS ONE. The reviewers have provided detailed feedback and highlighted a number of issues that require your attention. We kindly ask that you address these comments carefully in your revision.

We look forward to reviewing your updated manuscript.

[Note: HTML markup is below. Please do not edit.]

Reviewers' comments:

Reviewer's Responses to Questions

**Comments to the Author**

1. Is the manuscript technically sound, and do the data support the conclusions?

The manuscript must describe a technically sound piece of scientific research with data that supports the conclusions. Experiments must have been conducted rigorously, with appropriate controls, replication, and sample sizes. The conclusions must be drawn appropriately based on the data presented.

Reviewer #1: Partly

Reviewer #2: Partly

Reviewer #3: Yes

Reviewer #4: Partly

**********

2. Has the statistical analysis been performed appropriately and rigorously?

Reviewer #1: Yes

Reviewer #2: Yes

Reviewer #3: Yes

Reviewer #4: Yes

**********

3. Have the authors made all data underlying the findings in their manuscript fully available?

The PLOS Data policy requires authors to make all data underlying the findings described in their manuscript fully available without restriction, with rare exception (please refer to the Data Availability Statement in the manuscript PDF file). The data should be provided as part of the manuscript or its supporting information, or deposited to a public repository. For example, in addition to summary statistics, the data points behind means, medians and variance measures should be available. If there are restrictions on publicly sharing data—e.g. participant privacy or use of data from a third party—those must be specified.

Reviewer #1: No

Reviewer #2: Yes

Reviewer #3: Yes

Reviewer #4: Yes

**********

4. Is the manuscript presented in an intelligible fashion and written in standard English?

PLOS ONE does not copyedit accepted manuscripts, so the language in submitted articles must be clear, correct, and unambiguous. Any typographical or grammatical errors should be corrected at revision, so please note any specific errors here.

Reviewer #1: Yes

Reviewer #2: Yes

Reviewer #3: Yes

Reviewer #4: Yes

**********

5. Review Comments to the Author

Please use the space provided to explain your answers to the questions above. You may also include additional comments for the author, including concerns about dual publication, research ethics, or publication ethics. (Please upload your review as an attachment if it exceeds 20,000 characters)

Reviewer #1: Dear authors,

Thank you so much for writing manuscript in nursing student's attitude towards nursing research. The topic is very much evolving. However, go through the review attached in the pdf to make a robust manuscript.

Reviewer #2: 1.The research objective is to assess the attitude towards research, but the content seem to divert towards teaching and learning methods.

2. Did all of the students attend complete 14 weeks course?? were none of them absent??

Reviewer #3: • This study tests an important, but unproven hypothesis: whether the investment in a required research course leads to positive attitude changes in the classroom.

Title: (The current title is informative; however, consider whether a more active title would be more appropriate, for example, "Impact of a Research-Focused Intervention on Nursing Students' Attitudes...")

Introduction:

• The introduction follows a good funnel structure.

• The first paragraph lists studies reporting both positive and negative attitudes. It is recommended, however, to begin with a statement that directly links research to patient outcomes. Establishing the importance of nursing research and evidence-based practice (EBP) in the healthcare sector

o Example: "Evidence-based practice (EBP), supported by rigorous nursing research, provides high-quality, safe patient care and advances the nursing profession globally."

• The transition about cultural difference is a bit abrupt {line 84}. Integrate the context factors more smoothly

• In the second paragraph [line 86], effective strategies are listed. Provide a critical link to your study. You need to transition from this list of successful interventions to why your study remains important.

• The paragraph concerning faculty and course methods (beginning on line 98) seems disconnected. Reframe the final two paragraphs into one cohesive argument emphasizing the importance of pedagogical evidence.

• The final gap statement (line 104). "Additional research studies are required to determine the appropriate learning and teaching strategies...". Be sure to make your statement specific to the design of your study. The gap is significant and generalized. It should be specific to the intervention and location (KSA) you used.

• Study Aim. Make use of stronger, more active language (for an intervention study).

o Suggestion: "This study aimed to examine how a structured, credit-bearing undergraduate nursing research course influenced Saudi nursing students' attitudes toward nursing research, utilizing a pre-test/post-test quasi-experimental design."

Methods:

• It should be organized more clearly, characterized by a distinction between initial enrollment and final analysis.

• Specify the exact duration, the tools used, and the ethics approval number.

Results:

• Clarify the Sample Flow: Identify the final size of the analytic sample. Consider using a CONSORT/Flow Diagram concept (or explain the reason for exclusion) as soon as possible.

• Only the characteristics of the N=56 students used for the analysis are relevant to the results. To avoid redundancy, delete the initial N=62 description. In table 1, keep the data regarding the final sample (n=56).

• In table 1: Under the characteristics column, rephrase the question in shorter terms, for example: Were you oriented to research principles before taking this research course?” to [prior research course] and in the same way for the other questions.

• Is it the same six students who withdrew who did not complete the post-test?

• It is recommended that p-values be standardized by using a leading zero (e.g., p=0.01). Report results that are statistically significant up to two or three decimal places

• The report contains the mean difference and confidence interval (CI), which is excellent. For the paired t-test, however, a standardized effect size must be used (e.g., Cohen's d)

• Lines 210 to 224 of the report explain the overall significant finding, the two significant subscales, and the non-significant subscales, before repeating the detail for the two significant subscales. We recommend not repeating the detailed description.

• There are four subscales on your questionnaire: Research Abilities, Using Research in Clinical Practice, Personal Interest in Research, and Usefulness of Research. Please ensure that these exact and consistent names are used throughout the entire document.

• In line 228-233, the reporting correlation is r=-0.31, p=0.02. Although this is correct, it does not provide the full context. Be sure to use correct notation and interpretation: Identify the full test (e.g., Pearson's r), the correlation coefficient (r), the degrees of freedom (N-2), and the p-value.

• Interpreting GPA correlations. It should be noted that the finding is a significant inverse correlation (r = -0.33, p = 0.01). It is a very interesting, non-obvious finding that should be emphasized clearly.

o Example: "A significant inverse correlation was observed between GPA and total pre-test attitudes (r(54)=-0.33, p=0.01), suggesting that students with higher academic achievement reported lower attitudes toward research."

• The post-test correlation is missing. Only pre-test correlations are included in the report. Were these characteristics correlated with post-test scores or did they affect scores?

Discussion

• The discussion should be even more structured, critical, and focused on linking the specific components of your intervention to the observed attitudinal change.

• While the paragraphs flow well, the key components are mixed throughout.

• An overview of the key findings. This is covered in the first sentence, but it is weak. Prior to comparison with literature, a dedicated, clear summary statement is required.

• Include a paragraph dedicated to Interpretation of Specific Sub-scales, clearly linking the improvement to specific intervention strategies.

Linking Intervention to Results:

• Line 282-283: "The research course in the present study involved different strategies, such as mini-projects, critiquing articles, in-class teaching, and discussions". A discussion section would be better suited for a more specific description. It is essential to indicate which specific strategy led to the improvement in the significant domains.

• Paragraph starting at Line 251: "The findings contradict... Australia, which revealed no significant change... instruction was online, which differed from the method... This suggests a potential benefit of using interactive and in-class instruction...". The point is well taken, but it should be strengthened. Describe the difference in terms of a hypothesis for future research.

o Suggestion: "These conflicting outcomes underscore the importance of pedagogical modality; the non-significant findings in the Australian study, using online instruction (11), versus our face-to-face, interactive approach, suggest that direct student-instructor interaction may be a prerequisite to attitude change."

• In my opinion, the interpretation regarding GPA (academic pressure) is compelling. Be sure to specify that these are correlations with the pre-test score and that future studies should examine whether high-GPA students also show a lower rate of improvement (i.e., less course effect).

Reviewer #4: 1. Limitations of the Study Design and Causal Interpretation

The study employs a single-group pretest–posttest design without a control group. While this design is acknowledged in the limitations section, the manuscript occasionally uses language that implies a causal effect of the course on students’ attitudes (e.g., “effect,” “impact,” “improved by attending the course”).

Given the absence of a comparison group, alternative explanations such as maturation, concurrent coursework, or increased academic exposure over the semester cannot be ruled out.

Recommendations:

Revise wording throughout the Abstract, Discussion, and Conclusion to avoid causal implications.

Use more cautious language such as “was associated with,” “was followed by,” or “coincided with.”

Expand the discussion of potential confounding factors that could have contributed to changes in attitudes independent of the course itself.

2. Insufficient Detail on the Educational Intervention

Although the manuscript outlines the general content of the nursing research course, the description remains too broad to support reproducibility or meaningful comparison with other educational interventions.

Recommendations:

Clarify which learning activities were mandatory for all students (e.g., mini-projects, article critiques, oral presentations).

Specify how student performance was assessed and whether research-related activities contributed to course grades.

Consider providing a concise week-by-week overview or course framework, possibly as supplementary material.

3. Statistical Analysis and Interpretation of Effect Size

The authors appropriately report paired t-test results and Cohen’s d. However, the interpretation of the effect size is limited.

The reported Cohen’s d of 0.34 indicates a small to modest effect, which warrants a more nuanced discussion regarding its educational and practical significance.

Recommendations:

Elaborate on what a small-to-moderate effect size represents in the context of educational interventions.

Discuss whether the observed changes are likely to be meaningful in real-world educational or clinical settings.

Consider reporting and interpreting change scores or confidence intervals more explicitly to strengthen the analytical narrative.

4. Interpretation of the Negative Association Between GPA and Research Attitudes

The finding that higher GPA is negatively correlated with attitudes toward research is potentially interesting, but the explanation provided is largely speculative.

Recommendations:

Clearly distinguish empirical findings from interpretative hypotheses.

Acknowledge this result as exploratory rather than definitive.

Consider alternative explanations, such as academic pressure, assessment structure, or sampling characteristics, and explicitly state that further research is needed to clarify this relationship.

5.Minor Comments

There is an inconsistency in the reported SPSS version (version 23 in the Methods section versus version 26 in the references); please standardize.

In Table 2, the column labeled “d” refers to degrees of freedom rather than effect size, which may confuse readers. Please revise the label or clarify in the table note.

Minor grammatical and stylistic inconsistencies are present�British vs. American spelling; a careful language edit is recommended.

The Introduction could be slightly condensed to improve focus on the specific research gap addressed by the study.

**********

6. PLOS authors have the option to publish the peer review history of their article (what does this mean?). If published, this will include your full peer review and any attached files.

If you choose “no”, your identity will remain anonymous but your review may still be made public.

**Do you want your identity to be public for this peer review?**  For information about this choice, including consent withdrawal, please see our Privacy Policy.

Reviewer #1: **Yes:**  Bidhya Banstola

Reviewer #2: **Yes:**  Ms. Subina Bajracharya

Reviewer #3: **Yes:**  Rasha Dabbour

Reviewer #4: No

**********

[NOTE: If reviewer comments were submitted as an attachment file, they will be attached to this email and accessible via the submission site. Please log into your account, locate the manuscript record, and check for the action link "View Attachments". If this link does not appear, there are no attachment files.]

To ensure your figures meet our technical requirements, please review our figure guidelines: https://journals.plos.org/plosone/s/figures

You may also use PLOS’s free figure tool, NAAS, to help you prepare publication quality figures: https://journals.plos.org/plosone/s/figures#loc-tools-for-figure-preparation.

NAAS will assess whether your figures meet our technical requirements by comparing each figure against our figure specifications.

Attachment Submitted filename: PONE-D-25-61156_reviewed on 4th Dec.pdf

Attachment Submitted filename: PONE-D-25-61156_reviewer_comment_RD_17DEC2025.docx

# Author comment: Author response to Decision Letter 1

**DOI:** 10.1371/journal.pone.0351538.r002

25 Feb 2026

We sincerely thank the Editor and all reviewers for their thorough evaluation of our manuscript and for the constructive comments provided. All comments were carefully addressed, and the manuscript was revised accordingly to improve methodological rigour, clarity, and overall quality. All changes made in response to the reviewers’ comments are highlighted in light blue in the revised manuscript. Below, we provide a detailed, point-by-point response.

Response to Editor

Attachment Submitted filename: Response to reviewers PONE-D-25-61156.docx

# Peer review: Decision Letter 1

**DOI:** 10.1371/journal.pone.0351538.r003

**Authors:** Ahmed El-Sayed, Ahmed El-Sayed

22 Mar 2026

PONE-D-25-61156R1

Does Attending a Structured Undergraduate Nursing Research Course Affect Nursing Students’ Attitudes toward Nursing Research? A Quasi-experimental Study

PLOS One

Dear Dr. AL-Mugheed,

Thank you for submitting your manuscript to PLOS ONE. After careful consideration, we feel that it has merit but does not fully meet PLOS ONE’s publication criteria as it currently stands. Therefore, we invite you to submit a revised version of the manuscript that addresses the points raised during the review process.

Please submit your revised manuscript by May 06 2026 11:59PM. If you will need more time than this to complete your revisions, please reply to this message or contact the journal office at plosone@plos.org. When you're ready to submit your revision, log on to https://www.editorialmanager.com/pone/ and select the 'Submissions Needing Revision' folder to locate your manuscript file.

Please include the following items when submitting your revised manuscript:

- A letter that responds to each point raised by the academic editor and reviewer(s). You should upload this letter as a separate file labeled 'Response to Reviewers'.
- A marked-up copy of your manuscript that highlights changes made to the original version. You should upload this as a separate file labeled 'Revised Manuscript with Track Changes'.
- An unmarked version of your revised paper without tracked changes. You should upload this as a separate file labeled 'Manuscript'.

-->If you would like to make changes to your financial disclosure, please include your updated statement in your cover letter. Guidelines for resubmitting your figure files are available below the reviewer comments at the end of this letter.

If applicable, we recommend that you deposit your laboratory protocols in protocols.io to enhance the reproducibility of your results. Protocols.io assigns your protocol its own identifier (DOI) so that it can be cited independently in the future. For instructions see: https://journals.plos.org/plosone/s/submission-guidelines#loc-laboratory-protocols. Additionally, PLOS ONE offers an option for publishing peer-reviewed Lab Protocol articles, which describe protocols hosted on protocols.io. Read more information on sharing protocols at https://plos.org/protocols?utm_medium=editorial-email&utm_source=authorletters&utm_campaign=protocols.

We look forward to receiving your revised manuscript.

Kind regards,

Ahmed Abdelwahab Ibrahim El-Sayed

Academic Editor

PLOS One

Journal Requirements:

1. If the reviewer comments include a recommendation to cite specific previously published works, please review and evaluate these publications to determine whether they are relevant and should be cited. There is no requirement to cite these works unless the editor has indicated otherwise.

2. Please review your reference list to ensure that it is complete and correct. If you have cited papers that have been retracted, please include the rationale for doing so in the manuscript text, or remove these references and replace them with relevant current references. Any changes to the reference list should be mentioned in the rebuttal letter that accompanies your revised manuscript. If you need to cite a retracted article, indicate the article’s retracted status in the References list and also include a citation and full reference for the retraction notice.

**Additional Editor Comments:**

Dear Authors,

Thank you for submitting the revised version of your manuscript. I have now completed my review and would like to acknowledge the effort you have invested in addressing the previous comments.

However, there are still several minor issues that require careful attention before a final decision can be made. These points have been raised in detail by the reviewer and should be addressed thoroughly to ensure the manuscript meets the required standards.

In addition, the manuscript requires extensive language editing and refinement. At its current stage, it is not suitable for publication unless these revisions are carried out carefully and comprehensively.

Furthermore, please clearly disclose any use of AI tools in the preparation of this manuscript. There are sections of the text that appear to be AI-generated, and transparency in this regard is essential in accordance with publication ethics.

I encourage you to revise the manuscript accordingly and resubmit once all concerns have been properly addressed.

Reviewers' comments:

Reviewer's Responses to Questions

**Comments to the Author**

1. If the authors have adequately addressed your comments raised in a previous round of review and you feel that this manuscript is now acceptable for publication, you may indicate that here to bypass the “Comments to the Author” section, enter your conflict of interest statement in the “Confidential to Editor” section, and submit your "Accept" recommendation.

Reviewer #1: All comments have been addressed

Reviewer #2: All comments have been addressed

Reviewer #3: All comments have been addressed

**********

2. Is the manuscript technically sound, and do the data support the conclusions?

The manuscript must describe a technically sound piece of scientific research with data that supports the conclusions. Experiments must have been conducted rigorously, with appropriate controls, replication, and sample sizes. The conclusions must be drawn appropriately based on the data presented.

Reviewer #1: Partly

Reviewer #2: Yes

Reviewer #3: Yes

**********

3. Has the statistical analysis been performed appropriately and rigorously?

Reviewer #1: Yes

Reviewer #2: Yes

Reviewer #3: Yes

**********

4. Have the authors made all data underlying the findings in their manuscript fully available?

The PLOS Data policy requires authors to make all data underlying the findings described in their manuscript fully available without restriction, with rare exception (please refer to the Data Availability Statement in the manuscript PDF file). The data should be provided as part of the manuscript or its supporting information, or deposited to a public repository. For example, in addition to summary statistics, the data points behind means, medians and variance measures should be available. If there are restrictions on publicly sharing data—e.g. participant privacy or use of data from a third party—those must be specified.

Reviewer #1: Yes

Reviewer #2: Yes

Reviewer #3: Yes

**********

5. Is the manuscript presented in an intelligible fashion and written in standard English?

PLOS ONE does not copyedit accepted manuscripts, so the language in submitted articles must be clear, correct, and unambiguous. Any typographical or grammatical errors should be corrected at revision, so please note any specific errors here.

Reviewer #1: Yes

Reviewer #2: Yes

Reviewer #3: Yes

**********

6. Review Comments to the Author

Please use the space provided to explain your answers to the questions above. You may also include additional comments for the author, including concerns about dual publication, research ethics, or publication ethics. (Please upload your review as an attachment if it exceeds 20,000 characters)

Reviewer #1: Dear authors,

Please rewrite the title in statement form as that of short title but it would not be better to state it as "Impact " as the findings is just associated but has not established the temporal relationship and also add the population.

In the result section, n=56 so the findings will be of 56 only not for 62.

The correlation can be better displayed through plots rather than text results.

please mention that the 14 weeks' intervention has been measured only after 5 months and this causes problems in presenting your results just because of your intervention. The posttest findings are contaminated. It would have been much better if you have included other standarization of mesurement of variables such as research critique practice, clinical skills measurement techniques etc. You have mentioned as you have standarized but also mention in methodlogy section about how did you standarize? You have highlighted such teaching learning methodologies in literature gaps but not included in your study as a tool. You can keep it as a limitations of your study and further recommendations for other researchers.

Reviewer #2: All the comments have been addressed.

It would have been better to mention whether the 14 weeks course included

any hands-on training sessions.

Were the students involved in any actual research project during the course?

Reviewer #3: (No Response)

**********

7. PLOS authors have the option to publish the peer review history of their article (what does this mean?). If published, this will include your full peer review and any attached files.

If you choose “no”, your identity will remain anonymous but your review may still be made public.

**Do you want your identity to be public for this peer review?**  For information about this choice, including consent withdrawal, please see our Privacy Policy.

Reviewer #1: **Yes:**  Bidhya Banstola

Reviewer #2: No

Reviewer #3: **Yes:**  Rasha Dabbour

**********

[NOTE: If reviewer comments were submitted as an attachment file, they will be attached to this email and accessible via the submission site. Please log into your account, locate the manuscript record, and check for the action link "View Attachments". If this link does not appear, there are no attachment files.]

To ensure your figures meet our technical requirements, please review our figure guidelines: https://journals.plos.org/plosone/s/figures

You may also use PLOS’s free figure tool, NAAS, to help you prepare publication quality figures: https://journals.plos.org/plosone/s/figures#loc-tools-for-figure-preparation.

NAAS will assess whether your figures meet our technical requirements by comparing each figure against our figure specifications.

# Author comment: Author response to Decision Letter 2

**DOI:** 10.1371/journal.pone.0351538.r004

6 Apr 2026

all comment were revised

Attachment Submitted filename: Response_to_reviewers_PONE-D-25-61156_auresp_2.docx

# Peer review: Decision Letter 2

**DOI:** 10.1371/journal.pone.0351538.r005

**Authors:** Ahmed El-Sayed, Ahmed El-Sayed, Othman Alfuqaha

28 May 2026

Does attending a structured undergraduate nursing research course affect nursing students’ attitudes toward nursing research? A quasi-experimental study

PONE-D-25-61156R2

Dear Dr. Khalid AL-Mugheed,

We’re pleased to inform you that your manuscript has been judged scientifically suitable for publication and will be formally accepted for publication once it meets all outstanding technical requirements.

Within one week, you’ll receive an e-mail detailing the required amendments. When these have been addressed, you’ll receive a formal acceptance letter and your manuscript will be scheduled for publication.

An invoice will be generated when your article is formally accepted. Please note, if your institution has a publishing partnership with PLOS and your article meets the relevant criteria, all or part of your publication costs will be covered. Please make sure your user information is up-to-date by logging into Editorial Manager at Editorial Manager® and clicking the ‘Update My Information' link at the top of the page. For questions related to billing, please contact billing support.

If your institution or institutions have a press office, please notify them about your upcoming paper to help maximize its impact. If they’ll be preparing press materials, please inform our press team as soon as possible -- no later than 48 hours after receiving the formal acceptance. Your manuscript will remain under strict press embargo until 2 pm Eastern Time on the date of publication. For more information, please contact onepress@plos.org.

Kind regards,

Othman A. Alfuqaha, Ph.D.

Academic Editor

PLOS One

Additional Editor Comments (optional):

As the Academic Editor and based on the reviewers' recommendations, I am pleased to inform you that your manuscript has been accepted for publication.

Reviewers' comments:

Reviewer's Responses to Questions

**Comments to the Author**

1. If the authors have adequately addressed your comments raised in a previous round of review and you feel that this manuscript is now acceptable for publication, you may indicate that here to bypass the “Comments to the Author” section, enter your conflict of interest statement in the “Confidential to Editor” section, and submit your "Accept" recommendation.

Reviewer #2: All comments have been addressed

Reviewer #3: All comments have been addressed

**********

2. Is the manuscript technically sound, and do the data support the conclusions?

The manuscript must describe a technically sound piece of scientific research with data that supports the conclusions. Experiments must have been conducted rigorously, with appropriate controls, replication, and sample sizes. The conclusions must be drawn appropriately based on the data presented.

Reviewer #2: Yes

Reviewer #3: Yes

**********

3. Has the statistical analysis been performed appropriately and rigorously?

Reviewer #2: Yes

Reviewer #3: Yes

**********

4. Have the authors made all data underlying the findings in their manuscript fully available?

The PLOS Data policy requires authors to make all data underlying the findings described in their manuscript fully available without restriction, with rare exception (please refer to the Data Availability Statement in the manuscript PDF file). The data should be provided as part of the manuscript or its supporting information, or deposited to a public repository. For example, in addition to summary statistics, the data points behind means, medians and variance measures should be available. If there are restrictions on publicly sharing data—e.g. participant privacy or use of data from a third party—those must be specified.

Reviewer #2: Yes

Reviewer #3: Yes

**********

5. Is the manuscript presented in an intelligible fashion and written in standard English?

PLOS ONE does not copyedit accepted manuscripts, so the language in submitted articles must be clear, correct, and unambiguous. Any typographical or grammatical errors should be corrected at revision, so please note any specific errors here.

Reviewer #2: Yes

Reviewer #3: Yes

**********

6. Review Comments to the Author

Please use the space provided to explain your answers to the questions above. You may also include additional comments for the author, including concerns about dual publication, research ethics, or publication ethics. (Please upload your review as an attachment if it exceeds 20,000 characters)

Reviewer #2: (No Response)

Reviewer #3: (No Response)

**********

7. PLOS authors have the option to publish the peer review history of their article (what does this mean?). If published, this will include your full peer review and any attached files.

If you choose “no”, your identity will remain anonymous but your review may still be made public.

**Do you want your identity to be public for this peer review?**  For information about this choice, including consent withdrawal, please see our Privacy Policy.

Reviewer #2: **Yes:**  Subina Bajracharya

Reviewer #3: **Yes:**  Rasha Dabbour

**********

# Editor report: Acceptance letter

**DOI:** 10.1371/journal.pone.0351538.r006

**Authors:** Ahmed El-Sayed, Ahmed El-Sayed, Othman Alfuqaha

PONE-D-25-61156R2

PLOS One

Dear Dr. AL-Mugheed,

I'm pleased to inform you that your manuscript has been deemed suitable for publication in PLOS One. Congratulations! Your manuscript is now being handed over to our production team.

At this stage, our production department will prepare your paper for publication. This includes ensuring the following:

* All references, tables, and figures are properly cited

* All relevant supporting information is included in the manuscript submission,

* There are no issues that prevent the paper from being properly typeset

You will receive further instructions from the production team, including instructions on how to review your proof when it is ready. Please keep in mind that we are working through a large volume of accepted articles, so please give us a few days to review your paper and let you know the next and final steps.

Lastly, if your institution or institutions have a press office, please let them know about your upcoming paper now to help maximize its impact. If they'll be preparing press materials, please inform our press team within the next 48 hours. Your manuscript will remain under strict press embargo until 2 pm Eastern Time on the date of publication. For more information, please contact onepress@plos.org.

You will receive an invoice from PLOS for your publication fee after your manuscript has reached the completed accept phase. If you receive an email requesting payment before acceptance or for any other service, this may be a phishing scheme. Learn how to identify phishing emails and protect your accounts at https://explore.plos.org/phishing.

If we can help with anything else, please email us at customercare@plos.org.

Thank you for submitting your work to PLOS ONE and supporting open access.

Kind regards,

PLOS ONE Editorial Office Staff

on behalf of

Dr. Othman A. Alfuqaha

Academic Editor

PLOS One
